# Drought tolerance classification using unmanned aerial systems based on RGB and multispectral data

**DOI:** 10.3389/fpls.2026.1853372

**Published:** 2026-06-29

**Authors:** Helcio Duarte Pereira, Juliana Vieira Almeida Nonato, Rafaela Caroline Rangni Moltocaro Duarte, Isabel Rodrigues Gerhardt, Ricardo Augusto Dante, Juliana Erika de Carvalho Teixeira Yassitepe

**Affiliations:** 1 Genomics for Climate Change Research Center (GCCRC), Campinas, Brazil; 2 Centro de Biologia Molecular e Engenharia Genética (CBMEG), Campinas, Brazil; 3 Embrapa Meio Ambiente, Jaguariúna, Brazil; 4 Embrapa Agricultura Digital, Campinas, Brazil

**Keywords:** machine learning, multiple field trait evaluations, NDVI, NIR, plant breeding, unmanned aerial vehicle (UAV), *Zea mays* L.

## Abstract

Drought poses a global threat to food security and demands intensified efforts from breeding programs. Yet the lack of efficient methods for selecting this trait increases the cost and time required to develop new cultivars. The goal of this work was to assess the feasibility of using spectral data from RGB or multispectral sensors for drought-tolerance classification across various machine-learning models under the most practical cross-validation scenarios typical in breeding programs. The genotypes were assessed during trials conducted under either optimal (irrigated) or drought-stress conditions across two years, and evaluated using up to 10 field traits to determine their drought-tolerance classification based on membership function values related to drought. RGB and multispectral vegetation indices collected during several flights throughout the crop cycle were used to train machine learning models. We found that drought trials offer the best training data. Specificity was the metric most affected by sensor type and the nature of the training data. The multispectral sensor outperformed the RGB sensor on most evaluation metrics in both years. AdaBoost and linear discriminant analysis models demonstrated the strongest consistency across all prediction scenarios. Together, they achieved an overall accuracy, specificity, and F1-Score of 0.71, 0.56, and 0.77, respectively. The most influential vegetation indices for model performance consistently included the NIR band. Spectral information, such as vegetation indices, is a useful tool for plant researchers to complement drought tolerance evaluations in the field. This data-driven approach facilitates automation, paving the way to speed genetic gains by including early assessments of drought tolerance in breeding pipeline, and improves resource utilization efficiency.

## 1 Introduction

Drought is the primary threat to agriculture, with its frequency and severity likely to increase under the current climate change scenario (Gebrechorkos et al., 2025; Li et al., 2023). Maize is the most produced grain crop worldwide, and Brazil ranks as the third-largest producer (FAO, 2024). Yield losses due to drought can be significant in this crop, reaching up to 85% (Su et al., 2019). In other crops the yield losses under drought stress are also expressive, like 34% in susceptible soybean lines (Chen et al., 2020), 23% in wheat (Al-Ashkar et al., 2021) and 62% in rice (Hallajian et al., 2024). Therefore, breeding programs recognize the importance of drought tolerance in developing new cultivars (Kim and Lee, 2023). However, this stress is not fully understood in crops (Sheoran et al., 2022), and its assessment, usually based on multiple traits or evaluations, can be costly and labor-intensive.

Including drought tolerance as a new trait for selection is not straightforward. Drought tolerance, unlike typical traits, isn’t directly measured; instead, it is inferred from related secondary traits, including yield and its components, morphological and growth traits, physiological and biochemical adjustments, visual assessments, and stress indices (Sun et al., 2021; Wen et al., 2023). There is no standard method for measuring water stress in plants, particularly under field conditions, where controlling confounding factors is challenging. The plant’s response to drought is complex and depends on the duration, intensity, and the specific stage of the plant’s life cycle at which the stress occurs (Jing et al., 2023). An additional complication is the timing of assessment, as stressful conditions lead to physiological and morphological changes in plants over time (Su et al., 2019). Consequently, reliable evaluation of plant tolerance should involve multiple assessments over time to form a comprehensive understanding.

Taken together, the complexity, temporal dynamics, and multi-trait nature of drought responses make field phenotyping both labor-intensive and costly, particularly when large populations are evaluated across environments. As a result, phenotyping has become a major bottleneck in breeding programs targeting drought tolerance. Unmanned aerial systems (UAS) offer promising alternatives for crop phenotyping due to their speed, objectivity, high-throughput capabilities, and potential for frequent data collection owing to their non-destructive nature (Bongomin et al., 2024; Hoyos-Villegas et al., 2025). The use of unmanned aerial vehicles (UAVs, or drones) equipped with imaging sensors has been a hot topic in plant stress phenotyping research recently (Kim et al., 2021; Sheoran et al., 2022). It offers high spatial and temporal resolutions at the plot level, which were not previously accessible via satellite imagery (Tao et al., 2022). Additionally, measuring time-sensitive traits reduces noise in plant responses by enabling faster evaluation (Hein et al., 2021; Makanza et al., 2018). Using images in plant phenotyping helps assess the plant’s physiological status at a specific moment. Variations in the structure and composition of plant tissues are reflected in differences in their spectra (Alves et al., 2024; Asaari et al., 2019; Lazarević et al., 2022; Ryckewaert et al., 2021; Wen et al., 2023). This has been studied extensively through satellite-based research, which predicts the impact of environmental stresses on crops using indices like NDVI (normalized difference vegetation index), a proxy for vegetation greenness closely linked to chlorophyll content and canopy structure (Li et al., 2023). High correlations between physiological traits and the reflectance of imagery-based traits (vegetation indices) have already been reported in the literature for sugarcane (stomatal conductance and chlorophyll content) by Khuimphukhieo et al. (2024); wheat (stomatal conductance and leaf water potential) by Antoniuk et al. (2023); two conifer species (foliar moisture content) by Lad et al. (2023); and maize (stomatal conductance and leaf area index) by Zhang et al. (2021). Spectral reflectance data effectively classify drought-tolerant wheat genotypes when compared with a set of yield and physiological traits using multivariate analysis (Wen et al., 2023).

Image-based high-throughput phenotyping, supported by machine learning methods, has been explored as a promising tool to assist plant stress research (Khanna et al., 2019; Lazarević et al., 2022; Nampally et al., 2023; Singh et al., 2016). More research in this field can clarify its effectiveness across a wider range of situations and, therefore, support broader application use. Additionally, creating effective methods to measure drought tolerance through images can enhance breeding programs, as this trait can be evaluated more quickly and affordably. Therefore, enhanced resource-use efficiency can offer further benefits, such as enabling larger trials, improving selection efficiency and accelerating genetic gain. This study aimed to assess whether spectral data from RGB or multispectral sensors could be effectively used to classify drought tolerance, employing various machine learning models in realistic cross-validation scenarios typical of breeding programs. Based on the assumption that plant response to environmental stresses could be captured by discriminative wavelengths, the specific objectives of this work were to i) identify the most suitable sensor and spectral index to label genotypes under contrasting environmental conditions; ii) evaluate how the classifier behaves with contrasting training data.

## 2 Materials and methods

### 2.1 Evaluations and experimental design

A total of 28 single-cross hybrids were evaluated in this study, under either optimal irrigation conditions or drought stress, during the 2023 and 2024 dry seasons (April–September) in Campinas, SP, Brazil (22° 54′ 23″ S, 47° 3′ 42″ W, 680 m above sea level; **Supplementary Table 1**). Water was supplied by an inline drip irrigation system until the plants reached the V6 stage (six fully expanded leaves with visible collar). After that, only the optimal irrigation treatment continued to receive water until the R5 stage (dent stage). The experiment used a randomized complete block design with three replications. Single-row plots measured 0.45 meters apart and were 5 meters long. Row and column information were recorded to account for spatial variability. Each trial consisted of seven ranges, with 12 plots per range. A set of ten morphological, cycle, and yield traits were evaluated in those field trials (**Table 1**).

**Table 1:** Field traits assessed in the trials along with their abbreviations used in this work, description of how they were evaluated and unit of measurement.

Trait	Abbreviation	Definition	Unit
Days to anthesis	DTA	The time when at least 50% of the plants in the plot had tassels	Days from sowing
Days to silking	DTS	The time when at least 50% of the plants in the plot first produced silk	Days from sowing
Anthesis-silking interval	ASI	The difference between DTS and DTA	Days
Plant height	PH	Distances from the soil to the flag leaf	Meter
Ear height	EH	Distances from the soil to the main ear insertion	Meter
Ear length	ED	Length of the ear from base to top	Centimeter
Ear diameter	EL	The width of the ear at its middle section	Centimeter
Ear index	EI	The ratio of the number of ears to the number of plants per plot	Dimensionless
Hundred grain weight	HGW	The average weight of 100 kernels from three samples per plot	Grams
Grain yield	GY	The total grain weight corrected for 14% grain moisture	t ha^-1^

In 2024, the traits EL, ED, and EI were not recorded. The remaining traits were measured as previously described. We did not perform any imputation of missing data for the analysis steps explained hereafter. Those phenotypic data were analyzed using a mixed-model framework to predict genetic values and variance components via an REML/BLUP approach in the lme4 R package (Bates et al., 2015). The following model was applied for each trial and year of evaluation.


$$Y_{ijkl}=\;\mu +\;G_{i}+\;L_{j}+\;R_{k}+\;B_{l}+\;\epsilon_{ijkl}$$

where $Y_{ijkl}$: vector of phenotypic records at plot level; μ: overall mean of the trait (constant of the model); $G_{i}$: random effect of the ith genotype with $G_{i}\;\frac{iid}{\sim }\;N(0,\;\sigma_{g}^{2})$, i∈ {1, 2, 3….28}; $L_{j}$: random effect of the jth row with $L_{j}\;\frac{iid}{\sim }\;N(0,\;\sigma_{l}^{2})$, j ∈ {1, 2, 3….12}; $R_{k}$: random effect of the kth range with $R_{k}\;\frac{iid}{\sim }\;N(0,\;\sigma_{r}^{2})$, k ∈ {1, 2, 3….7}; $B_{l}$: random effect of the lth block with $B_{l}\;\frac{iid}{\sim }\;N(0,\;\sigma_{b}^{2})$, l∈ {1, 2, 3}; and $\epsilon_{ijkl}$: random effect of residual for each observation with $\epsilon_{ijkl}\;\frac{iid}{\sim }\;N(0,\sigma_{e}^{2})$. The terms $\sigma_{g}^{2}$, $\sigma_{l}^{2}$, $\sigma_{r}^{2}$, $\sigma_{b}^{2}$ and $\sigma_{e}^{2}$ are variance components related to genotypes, rows, ranges, blocks and residuals, respectively. The genotype effect measures the genotypic variability of our population, the block effect informs about the environmental fluctuations among replications, whereas the row and range effects account for spatial variability of the experimental area in two directions.

The model’s random effects were tested using deviance analysis (Mrode, 2014). For this, nested models were constructed, meaning models with (full model) and without (reduced model) the random effect being tested. The specific random effect was evaluated using the likelihood ratio test at a 5% significance level. The R^2,^ representing the total variance explained by the model, was calculated using the conditional R^2^ approach for mixed models as described by Nakagawa and Schielzeth (2013). The heritability of the traits was determined as follows (Equation 1):

(1) $$\text{Heritability}=\;\frac{\;\sigma_{g}^{2}}{\;\sigma_{g}^{2}+\;\frac{\sigma_{e}^{2}}{number\;of\;replications}}$$

### 2.2 Drought tolerance classification

The BLUP predictions of genotypes were used in a unified approach to infer their drought tolerance using the membership function value to drought (MFVD) (Chen et al., 2012). First, for each genotype and trait, a drought coefficient is calculated with the genotype’s performance in irrigated and drought conditions, by Equation 2:

(2) $$DC_{ij}=\;\frac{Y_{ij\;(stress)}}{Y_{ij\;(control)}}$$

where $DC_{ij}$ is the drought coefficient for genotype i and trait j; $Y_{ij}$ is the BLUP prediction for genotype i and trait j. These coefficients are input values for a membership function, ranging from 0 (no membership) to 1 (full membership), for each trait. Thus, we get the membership function value for drought at the trait level (Equation 3):

(3) $$U_{ij}=\;\frac{DC_{ij}-\;DC_{j\;min}}{DC_{j\;max}-\;DC_{j\;min}}$$

where $U_{ij}$ is the membership function value for genotype i and trait j; $DC_{j\;min}$ and $DC_{j\;max}$ are the minimum and maximum drought coefficients found in population for trait j.

However, not all traits vary in the same direction, because for some of them increases under stress (DC>1) means lower membership, as ASI in our study. As the membership function in a fuzzy logic is limited to the closed interval [0, 1], for such category of traits we used a linear monotonically decreasing function (Equation 4):

(4) $$U_{ij}=\{\;\begin{array}{c} 1\\ \left(b-\;DC_{ij})/(b-a\right)\\ 0 \end{array}\;\begin{array}{l} if\;DC_{ij}\leq a\\ if\;a<DC_{ij}<b\\ if\;DC_{ij}\geq b \end{array}$$

where a is the lower threshold from which the membership starts decreasing and b is the upper threshold below which the membership increases. In this study the a was set as 1 because a DC for ASI above this value means that the genotype is suffering some level of drought stress, and below it the drought stress has no effect. The b was set as 2 because a DC for ASI above this value was very rarely observed in our work, then 2 was judged the upper threshold for drought stress (full stress). Any DC bellow a receive the highest membership grade of 1 (highest tolerance) and above b the lowest membership grade of 0 (lowest tolerance). Between a and b the membership decreases linearly from 1 to 0. The a and b were set as 1 and 2, respectively, in this study. All traits can be gathered together within each genotype to get an index of membership function value to drought (the higher the better) (Equation 5):

(5) $$U_{i}=\;\frac{1}{n}\;\overset{n}{\underset{j=1}{\sum }}U_{ij}$$

where $U_{i}$ is the average membership function value at the genotype level; n is the number of traits regarded.

Of the 28 genotypes, 21 were shared between the two years, while the other 7 were evaluated only in 2024. Consequently, the calculations of $U_{ij}$ and $U_{i}$ used different trait sets for these two groups. For the 21 common genotypes, we included all evaluations from 2023 and 2024 separately. For the remaining seven genotypes, only their 2024 evaluations were used. So, the first set had 21 genotypes evaluated for all ten traits in 2023 and seven traits in 2024. The second set had seven genotypes evaluated for seven traits in 2024. The genotypes were categorized into two groups - susceptible and tolerant - using the k-means clustering approach and the provided $U_{ij}$ and $U_{i}$.

### 2.3 UAV imaging and vegetation indices extraction

An unmanned aerial vehicle (UAV), a DJI Mavic 3M equipped with onboard RGB and multispectral sensors, was used to capture aerial images during the trials. The flights were conducted at 12 m height around noon (from 11 am to 1 pm) to ensure perpendicular solar incidence, on days with no rain or wind whenever possible. Images from both sensors were captured simultaneously on each flight. We set the frontal and side overlaps between sequential images to 90% and 80%, respectively. The equipment included a standard RGB sensor (5280 x 3956 pixels) and a multispectral sensor (2592 x 1944 pixels) with four channels: Green (560 ± 16 nm), Red (650 ± 16 nm), Red-Edge (730 ± 16 nm), and NIR (860 ± 26 nm).

**Figure 1:**
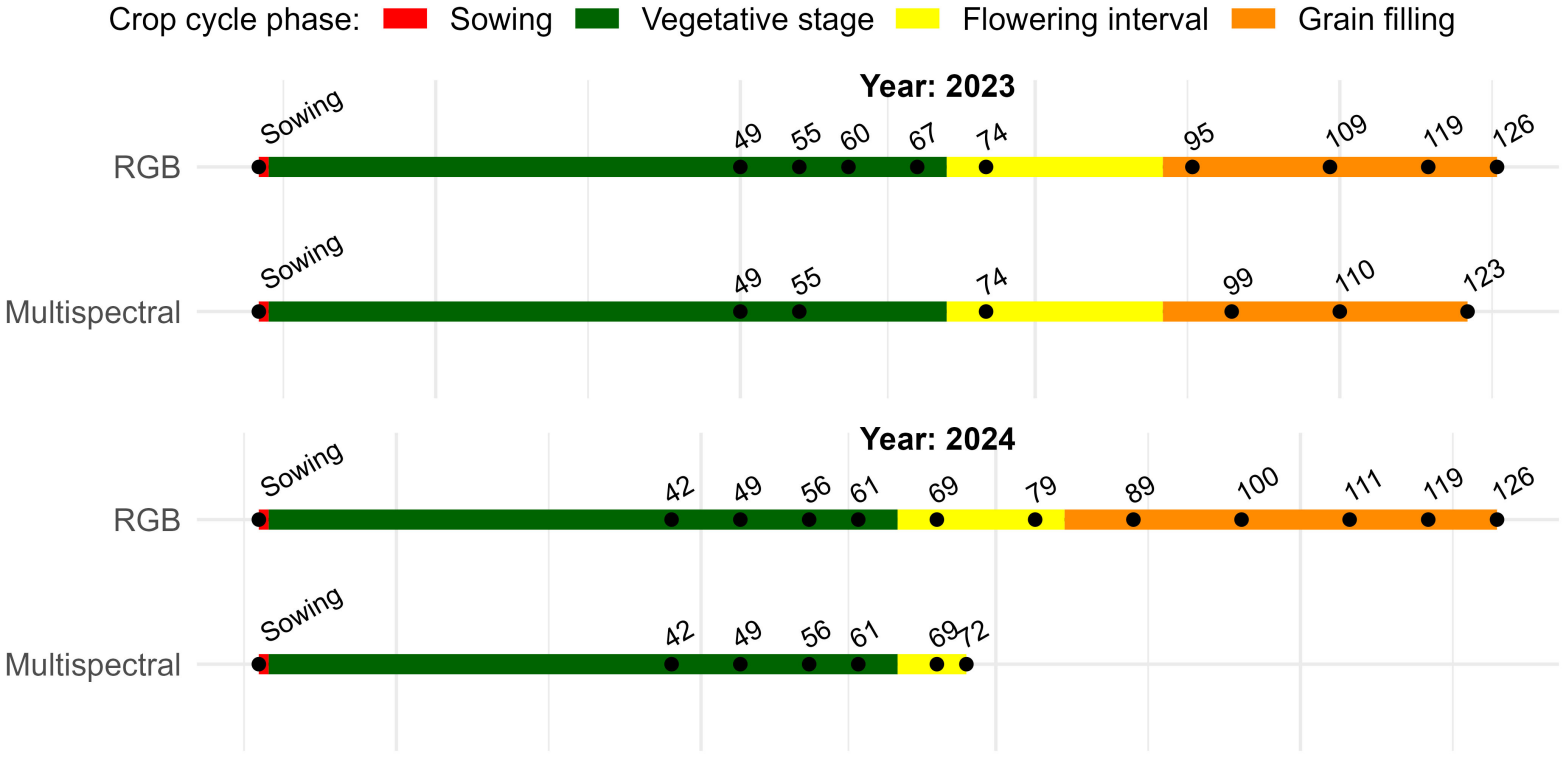
Flight dates by sensor for each year, expressed as days after planting (DAP), with corresponding crop phase.

The raw images were processed using Open Drone Map (via the WebODM interface), which stitched them into an orthomosaic. In the main user interface, we selected the options for *High Resolution* and *No Resize Images* for the RGB images, and radiometric calibration: *camera+sun* for the multispectral images. We chose the highest quality maps, free of shadows or blurring, to extract vegetation indices. Consequently, in 2023, we used nine RGB and six multispectral maps. In 2024, the selection included eleven RGB and six multispectral maps. These maps cover the entire crop cycle, from vegetative growth to grain filling, except for the 2024 multispectral maps, which were unavailable due to sensor technical issues (see **Figure 1**).

Next, we manually created the ESRI shapefiles (.shp) of the plots for each trial and flight date using QGIS (QGIS Development Team, 2024). To minimize interference between adjacent plots, we applied an 80% buffer using the Buffer by percentage plugin. The orthomosaics and polygons from the shapefiles were imported into R to extract plot-based vegetation indices. We developed an R script to clip each plot from the orthomosaic with the *terra* package (Hijmans, 2024) and the shapefile. The plants were segmented from the soil using a color mask with the field mask function of the *FIELDImageR* R package (Matias et al., 2020). The most effective vegetation indices for this segmentation were HUE and NDVI (threshold 0.6) for RGB and multispectral orthomosaics, respectively.

We calculated 35 and 54 vegetation indices from the three RGB bands and the four bands of the multispectral maps, respectively. The names of the vegetation indices, along with their formulas and references, are provided in **Supplementary Tables 2** and **S3** for each sensor. Bands from each pixel were used to calculate the vegetation index, and a trimmed mean - discarding the 10% extreme values - was used as the output at the plot level. This approach was taken to reduce the potential impact of noisy data in the segmented image.

These plot-level vegetation indices served as a new trait. Therefore, they were analyzed using a nested design that grouped all flights by sensor and trial to obtain temporal genetic values (TBLUPs) and variance components. The following model was used for each vegetation index:


$$Y_{ijklm}=\;\mu +F_{i}+\;G_{j(i)}+\;L_{k(i)}+\;R_{l(i)}+\;B_{m(i)}+\;\epsilon_{ijklm}$$

where $Y_{ijklm}$: record for vegetation index on each flight, given as DAP, at plot level; μ: overall mean; $F_{i}$: random effect of ith flight time with $F_{i}\;\frac{iid}{\sim }\;N(0,\;\sigma_{f}^{2})$, i ∈ {49, 55, 60, 67, 74, 95, 109, 119, 126} for RGB sensor in 2023, i ∈ {49, 55, 74, 99, 110, 123} for multispectral sensor in 2023, i ∈ {42, 49, 56, 61, 69, 79, 89, 100, 111, 119, 126} for RGB sensor in 2024; and i ∈ {42, 49, 56, 61, 69, 72} for multispectral sensor in 2024; $\;G_{j(i)}$: random effect of the jth genotype within the ith flight time with $G_{j(i)}\;\frac{iid}{\sim }\;N(0,\;\sigma_{g(f)}^{2})$, j ∈ {1, 2, 3….28}; $L_{k(i)}$: random effect of the kth row within the ith flight time with $L_{k(i)}\;\frac{iid}{\sim }\;N(0,\;\sigma_{l(f)}^{2})$, k ∈ {1, 2, 3….12}; $R_{l(i)}$: random effect of the lth range within the ith flight time with $R_{l(i)}\;\frac{iid}{\sim }\;N(0,\;\sigma_{r(f)}^{2})$, l ∈ {1, 2, 3….7}; $B_{m(i)}$: random effect of the mth block within the ith flight time with $B_{m(i)}\;\frac{iid}{\sim }\;N(0,\;\sigma_{b(f)}^{2})$, m ∈ {1, 2, 3}; and $\epsilon_{ijklm}$: random effect of residuals with $\epsilon_{ijklm}\;\frac{iid}{\sim }\;N(0,\;\sigma_{e}^{2})$. The terms $\sigma_{f}^{2}$, $\sigma_{g(f)}^{2}$, $\sigma_{l(f)}^{2}$, $\sigma_{r(f)}^{2}$, $\sigma_{b(f)}^{2},$ and $\sigma_{e}^{2}$ are variance components related to flights, genotypes, rows, ranges, blocks, and residuals, respectively.

**Figure 2:**
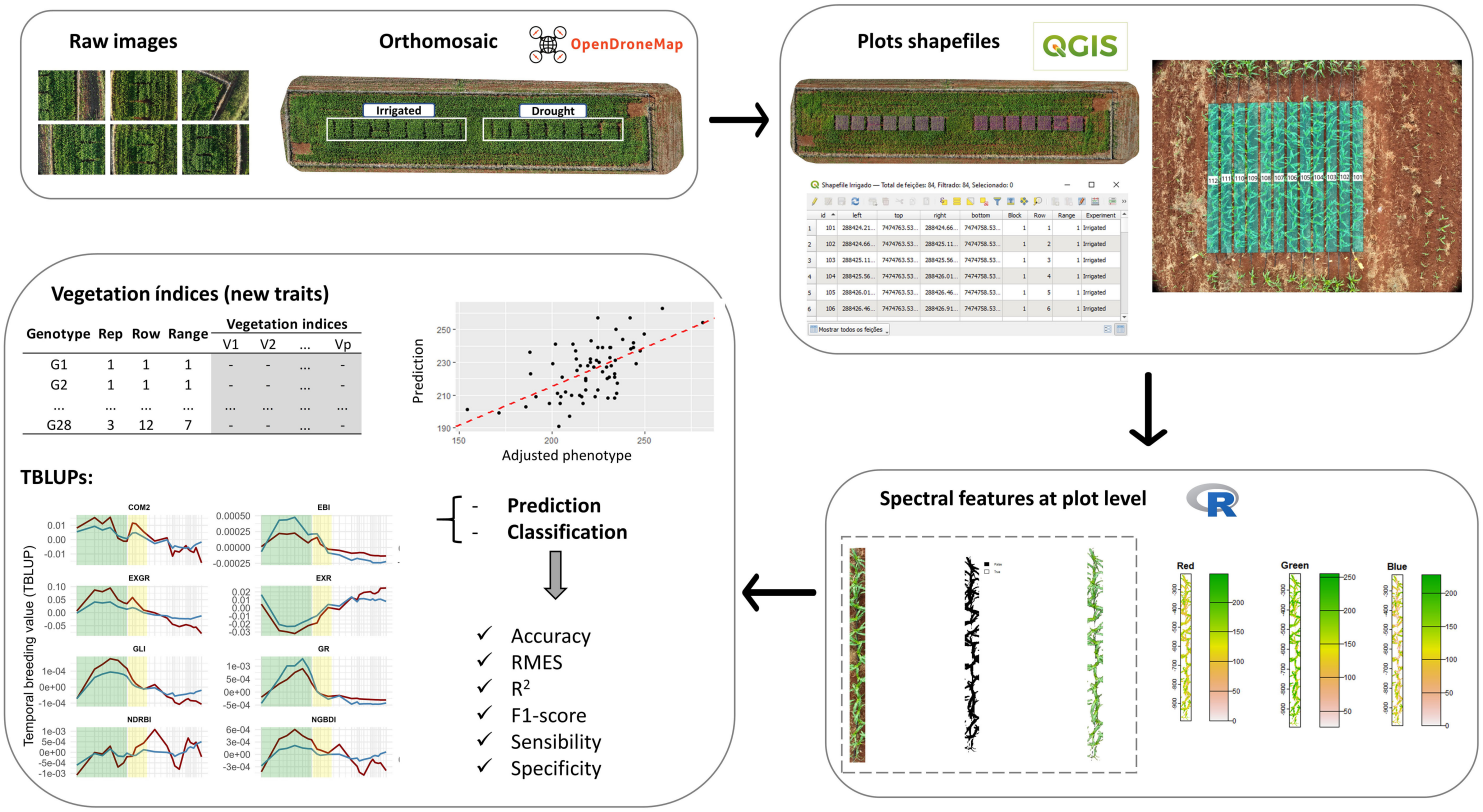
Pipeline of UAV imaging and phenotyping used in this work. The steps include constructing the orthomosaic from raw images, drawing plot shapefiles for each map, performing image segmentation and calculating vegetation indices, analyzing these vegetation indices as new traits, and applying them in high-throughput phenotyping protocols.

The R^2^ and significance of random effects were calculated as previously explained for the ground-based phenotypic traits. **Figure 2** illustrates the pipeline of UAV imaging and phenotyping by vegetation indices. Temporal repeatability of the genotypic effect of the index was calculated by Equation 6. Because we made many flights across the crop cycle, this repeatability measures the explained variance on the temporal scale.

(6) $$\text{Temporal Repeatability}=\;\frac{\;\sigma_{g(f)}^{2}}{\;\sigma_{g(f)}^{2}+\;\frac{\sigma_{e}^{2}}{number\;of\;replications}}$$

### 2.4 Vegetation indices, machine learning models, and genotype classification

The classification of genotypes for drought tolerance was also investigated using the genetic value of vegetation indices (TBLUP), as previously described. Only vegetation indices showing significant genotypic effects under drought conditions were included in the classification models. This preliminary selection aimed to reduce the computational burden and improve model performance. The number of predictors, specifically vegetation indices in our case, increases rapidly, as they depend on the number of flights multiplied by the number of vegetation indices used.

**Figure 3:**
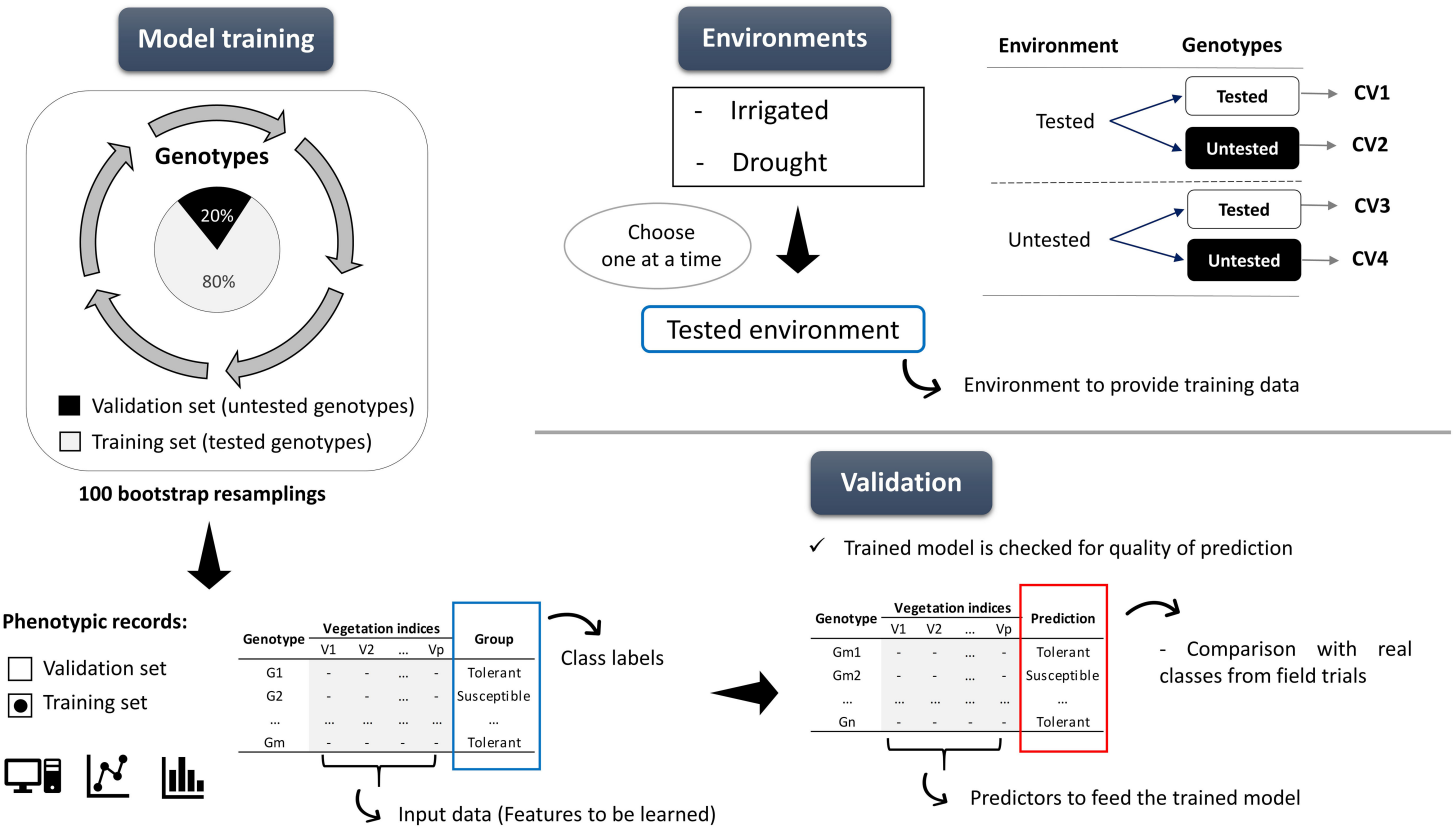
An iterative process involving model training and validation, illustrating how phenotypic data is used in supervised learning, along with cross-validation schemes (CV1, CV2, CV3, CV4) that combine environmental and genotype information.

We investigated some scenarios of interest for plant breeding using our classification approach, involving sets of tested and untested genotypes and environments. These can be understood as cross-validation scenarios: tested genotypes in a tested environment (CV1); untested genotypes in a tested environment (CV2); tested genotypes in an untested environment (CV3); and untested genotypes in an untested environment (CV4). CV1 serves as a benchmark for comparison. CV2 represents situations where the performance of new genotypes is unknown because they have not been tested in any environment. CV3 is similar to incomplete field trials, in which some genotypes are tested in certain environments but not in others. CV4 is the case where the goal is to determine the performance of new genotypes in environments where no phenotypic records have yet been collected. A total of 100 iterations were performed, and all of them were submitted to the four cross-validation scenarios. For each iteration, the genotypes were randomly split into two sets, 80 and 20%, which worked as training and validation genotypes, respectively. Our sampling process had the restriction that each set (training and validation) must include at least one genotype from each group (tolerant and susceptible). The training set comprised the genotypes used to train the models. After training, the validation set comprised untested genotypes used only for prediction, not for training. Both irrigated and drought environments served as tested and untested conditions, each applied separately (**Figure 3**).

We investigated eight machine learning models, differing in their properties and algorithms, for supervised drought-tolerance classification. The models were: AdaBoost, k-nearest neighbors, linear discriminant analysis, logistic regression, partial least squares, random forest, support vector machine with a linear kernel, and support vector machine with a radial kernel. These models were adjusted in the *caret* R package (Kuhn, 2008) using cross-validation with 10 resampling iterations (*numbers* = 10), repeated 3 times (*repeats* = 3), within the *trainControl* function. Each best model, that is, the one with the optimized parameters, was determined by maximizing the area under the *ROC* (Receiver Operating Characteristic) curve.

The tuning parameters for random forest were set as 500 *ntree* (number of trees) and a searched value between 1 and 30 for *mtry* (number of variables tested in each split). For the linear support vector machine, we defined *tuneGrid* to search values between 1 and 5, and for the radial support vector machine, we set *tuneLength* to 10. For partial least squares, we set *tuneLength* to 10 for the number of principal components. For the k-nearest neighbors, we set *tuneGrid* to search values between 3 and 20 in increments of 2. AdaBoost had a *tuneGrid* containing specifications of *mfinal* (number of trees), *maxdepth* (maximum depth of decision trees), and *coeflearn* (coefficient type or learning method). *mfinal* was set as a searched value between 3 and 15 in increments of 3; *maxdepth* was set as a searched value between 1 and 3 in increments of 1; and *coeflearn* was set to three options: *Breiman, Freund*, and *Zhu*. For logistic regression, the tuning parameters involve *alpha* (the mixing percentage of Lasso and Ridge regularization) and *lambda* (strength of the regularization penalty - shrinkage of coefficients). *alpha* was set as a searched value between 0 and 1 in ten equal increments, and *lambda* was set as a searched value between 0.0001 and 1 in ten equal increments.

The models were evaluated for their successes and failures by comparing predictions to the benchmark classification of traits assessed in field trials. Using the confusion matrix (**Supplementary Figure 1**), we calculated accuracy (the proportion of correct predictions) (Equation 7), sensitivity (recall) (Equation 8), precision (Equation 9), specificity (Equation 10), and F1-score (Equation 11). For all these metrics the positive case was the tolerant group and, hence, the negative the susceptible. Accuracy measures the proportion of correct classifications out of all cases. Sensitivity, or recall, measures the rate of true positives among actual positive cases. Precision measures the proportion of true positives among all predicted positives. Specificity measures the rate of true negatives among actual negative cases. The F1-score is the harmonic mean of sensitivity and precision. They are calculated as follows:

(7) $$accuracy=\;\frac{TP+TN}{TP+TN+FP+FN}$$

(8) $$sensitivity=\;\frac{TP}{TP+FN}$$

(9) $$precision=\;\frac{TP}{TP+FP}$$

(10) $$specificity=\;\frac{TN}{TN+FP}$$

(11) $$F1-score=\;\frac{2*sensitivity*precision}{sensitivity+\;precision}$$

where TP: true positives; TN: true negatives; FP: false positives; and FN: false negatives. The relative importance of predictors (scale of 0 - 100%) was recorded for each model and iteration.

## 3 Results

### 3.1 Field evaluations and drought tolerance classification

**Figure 4:**
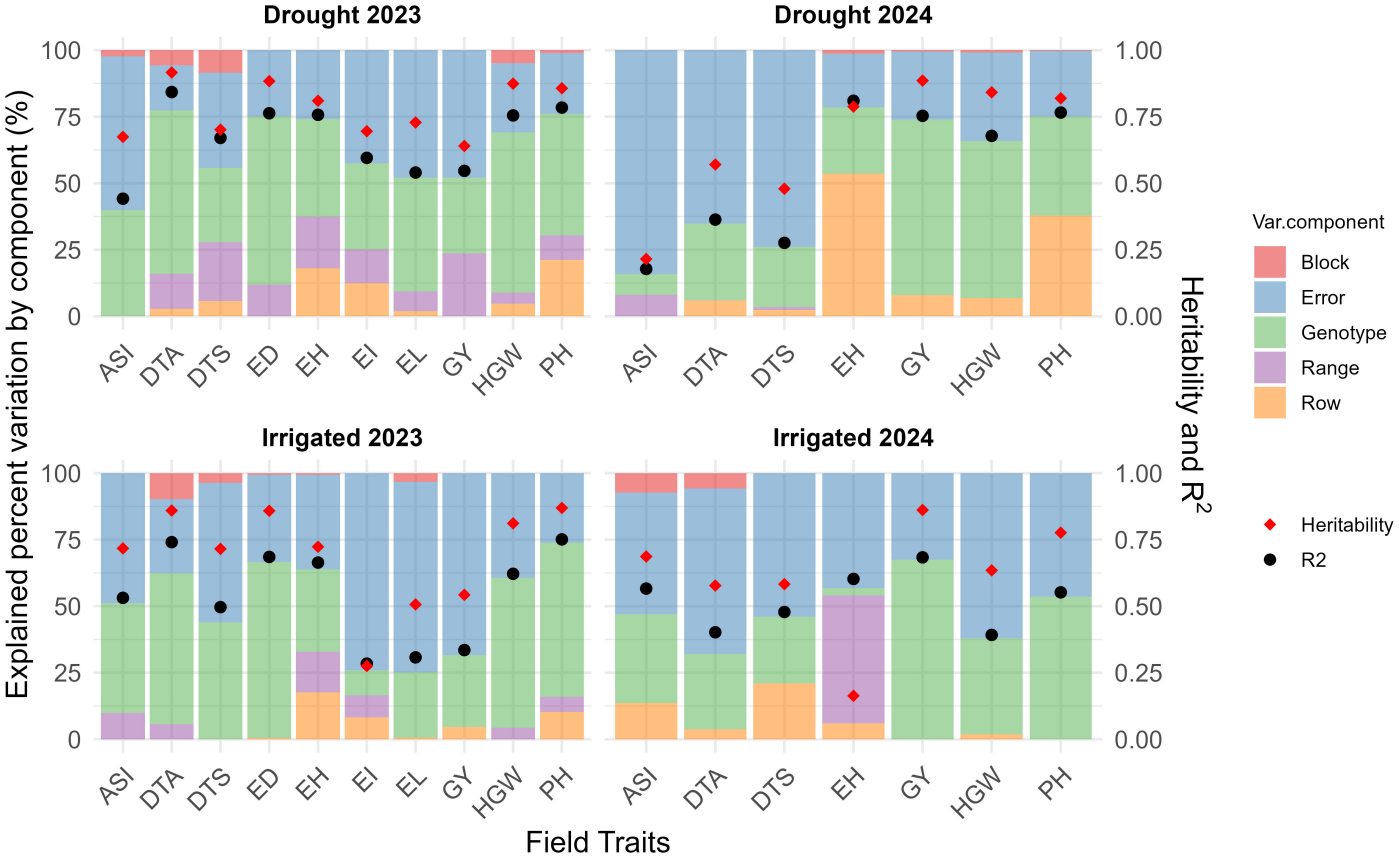
Relative proportions of variance components of random effects, R^2^ (the proportion of variance explained by the model), and trait heritability for each trial and year combination. The traits include: anthesis-silking interval (ASI), days to anthesis (DTA), days to silking (DTS), ear diameter (ED), ear height (EH), ear index (EI), ear length (EL), grain yield (GY), hundred grain weight (HGW), and plant height (PH). The left Y axis indicates the percentage of variance explained by each component, while the right Y axis displays heritability (red diamonds calculated with Equation 1) and R^2^ values (black round symbols).

The contribution of genotypic effects to the total variance was generally similar between drought and irrigated conditions across both years (**Figure 4**). However, the values were higher in 2023 (42.65%) compared to 2024 (35.22%). For R^2^ and heritability, the average values were also higher in 2023 than in 2024. Unlike the genotypic effects, these parameters did not behave the same within each condition. Drought trials showed higher R^2^ and heritability than irrigated ones, especially in 2023. This difference was much smaller in 2024. Across the trials, PH and HGW were the most consistent traits. EH also showed good consistency, except for the 2024 drought trial, where many data points were missing.

The performance of genotypes under drought conditions differed significantly from that under irrigated conditions in both years, with reductions of 43% and 31% in 2023 and 2024, respectively. The 2024 season experienced harsher climatic conditions, being drier and hotter than 2023, which was worsened by corn stunt disease. This disease caused even the irrigated trial results in 2024 to be far lower than those in 2023. A general overview of genotype performance across both years is shown in **Supplementary Figure 2**, which highlights the common traits observed.

For drought tolerance assessment, we excluded traits with many missing data and those with very little difference between the irrigated and drought performance, that is, with a drought coefficient (DC) very close to 1. The DC is the base statistic used to account for drought tolerance, based on the membership function value for drought. The **Supplementary Figures 3** and **S4** have the DC for all traits in 2023 and 2024, respectively. The DC of the following traits in 2023 were used: ASI, ED, EH, EL, GY, HGW, and PH. In 2024, the traits included were: ASI, GY, HGW, and PH. Across the entire dataset, genotypes were grouped into 17 tolerant and 11 susceptible. The values of the membership function for drought at the trait (U_ij_) and genotype level (U_i_) are shown in **Supplementary Table 4**. The tolerant group had an average U_i_ of 0.64, whereas the susceptible group had an U_i_ of 0.44.

### 3.2 Vegetation indices

**Figure 5:**
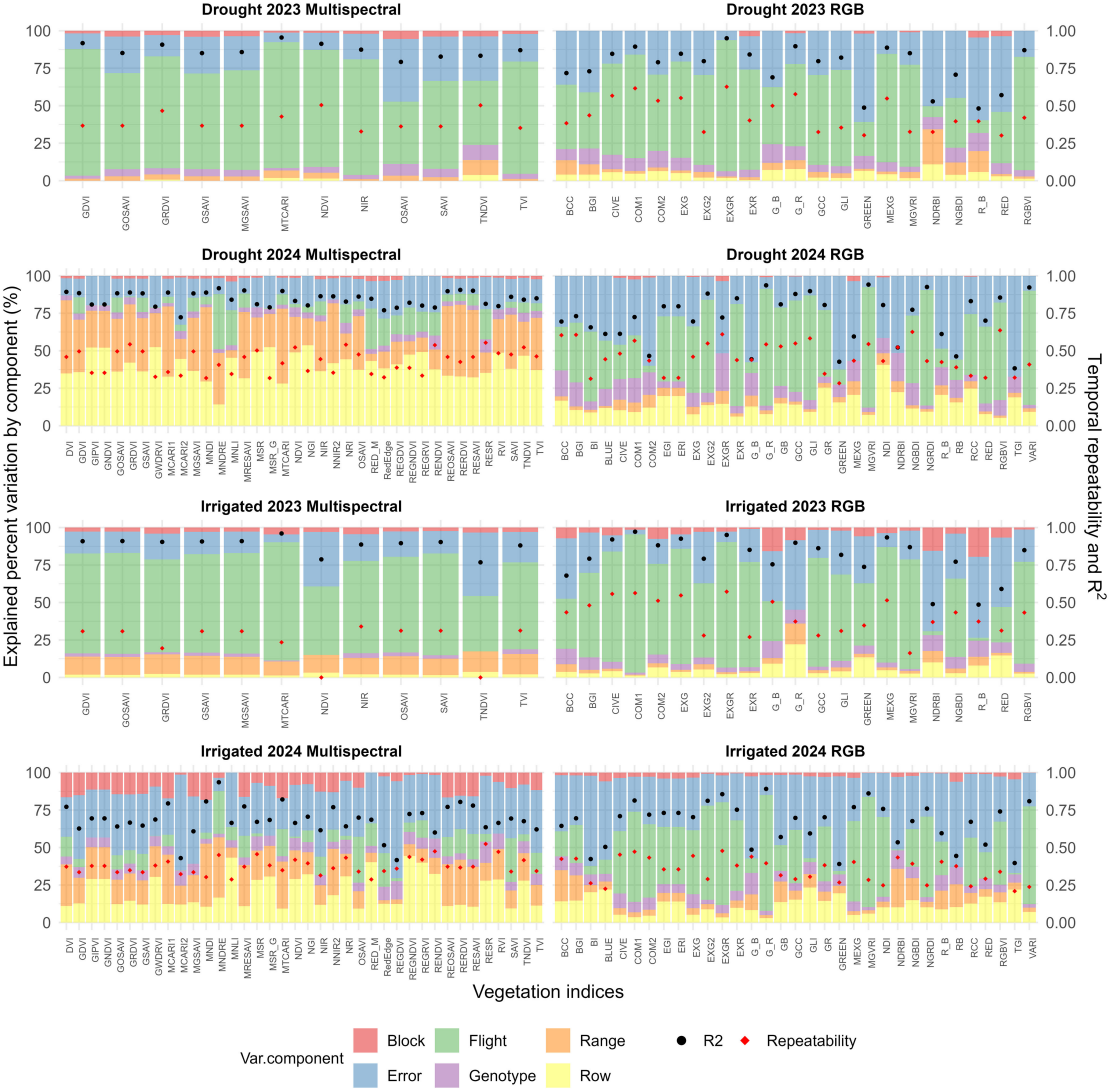
The relative proportion of variance components for random effects, R^2^ (the proportion of variance explained by the model), and the temporal repeatability of the indices for each combination of trial, year, and sensor. The left Y-axis represents the percentage of variation explained by the components, while the right Y-axis displays the temporal repeatability (red diamonds calculated by Equation 6) and R^2^ values (black round symbols).

Vegetation indices show considerable variation across different years, sensors, and drought scenarios (**Figure 5**). In 2023 and 2024, the RGB sensor identified 21 and 33 vegetation indices, respectively, that exhibited significant genotypic effects under drought conditions. The multispectral sensor detected 12 indices in 2023 and 38 in 2024 with similar genotypic effects. The impact of flight effects was notable across all combinations of sensors, years, and drought conditions, except for multispectral sensor indices in 2024, where flights did not cover the full crop cycle. The changes in the temporal genotypic effect of each vegetation index, comparing irrigated and drought conditions over time, are shown in **Supplementary Figures 5**–**8**, corresponding to the significant indices for RGB and multispectral sensors in 2023 and 2024.

The variance of the flight effect was similar between irrigated and drought conditions across both years and sensors, with higher variances observed for the multispectral sensor, especially in 2023. In 2024, this sensor was affected by fewer flights, resulting in a smaller flight-effect variance than RGB. Regarding the genotypic effect, variances between irrigated and drought trials were greater across years and sensors, with higher values for the RGB sensor. The R^2^ values and heritability of the vegetation indices were generally lower than those of the previously explained field traits. Although the indices exhibited higher R^2^, they tended to have lower heritability than the field traits in most cases.

### 3.3 Drought tolerance classification

This study explored various scenarios for drought-tolerance classification, including two training dataset sources, two sensors, four cross-validation scenarios, and eight machine learning models across two contrasting years, resulting in 256 configurations. The findings were carefully organized to highlight key insights. The main factor examined was the nature of the training data, specifically whether it originated under drought conditions. We found an overall advantage when models were trained with vegetation indices from drought trials. Across all models, sensors, years, and validation schemes, this advantage was approximately 8.47% in accuracy, 6.21% in precision, 5.05% in sensitivity, 20.96% in specificity, and 4.83% in F1-score. As a result, all subsequent results are based on models trained with drought trial data. Results for models trained with irrigated data are included in the **Supplementary Materials**.

**Figure 6:**
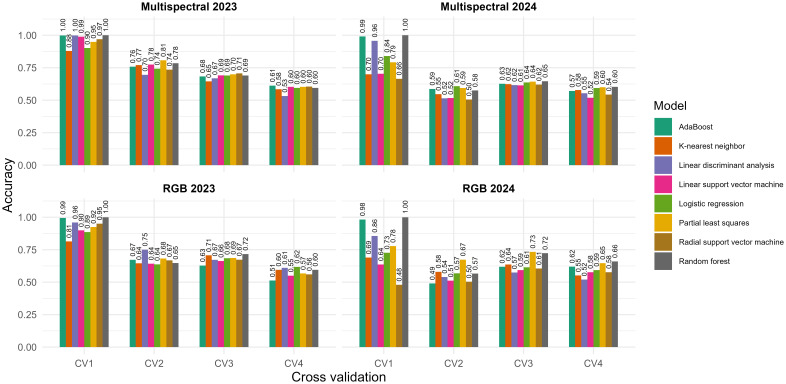
Accuracy of drought tolerance classification (Y axis) for each combination of sensor and year, alongside the four cross-validation schemes (X axis) and the eight machine learning models tested. CV1 and CV2 represent the classification of tested and untested genotypes in the observed environment (drought), respectively. CV3 and CV4 represent the classification of tested and untested genotypes in the unobserved environment (irrigated), respectively.

The accuracy (proportion of total concordance across all classifications) was higher in CV1 than in the other cross-validation scenarios, as expected (**Figure 6**). The models showed little difference among themselves within each cross-validation scenario and across sensor-year combinations, except in CV1 for both sensors in 2024. That year, the models AdaBoost, linear discriminant analysis, logistic regression, partial least squares, and random forest were notable for their performance (accuracy > 0.7). Overall, the multispectral sensor achieved higher accuracy than the RGB, especially in 2023 (by 5.01%) compared to 2024 (by 1.27%). The accuracy of model training in irrigated trials is detailed in **Supplementary Figure 9**.

**Figure 7:**
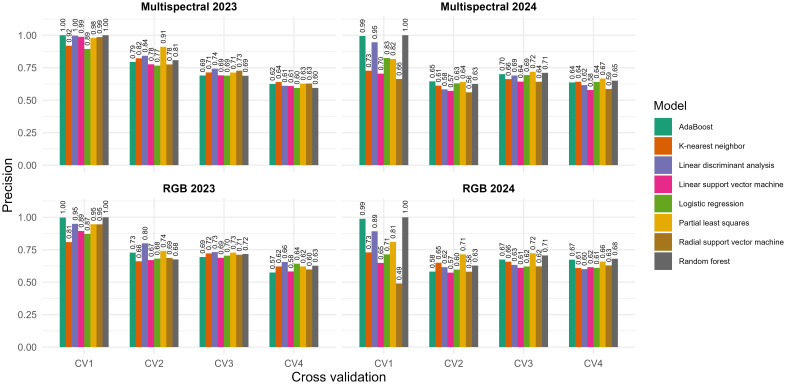
Precision of drought tolerance classification (Y axis) for each combination of sensor and year, across the four cross-validation schemes (X axis) and the eight machine learning models examined. CV1 and CV2 represent the classification of tested and untested genotypes in the observed environment (drought), respectively. CV3 and CV4 represent the classification of tested and untested genotypes in the unobserved environment (irrigated), respectively.

Precision (the proportion of classified tolerant genotypes that are truly tolerant) showed a pattern similar to accuracy, indicating that the models varied little among themselves within each cross-validation scenario and across sensor-year combinations, except for CV1 in 2024 for both sensors (**Figure 7**). Again, the models AdaBoost, linear discriminant analysis, logistic regression, partial least squares, and random forest were the most consistent, with precision > 0.7. On average, the multispectral sensor yielded higher precision than the RGB sensor. This advantage was approximately 4.97% in 2023 and 2.27% in 2024. The precisions obtained by training the models on irrigated trials are shown in **Supplementary Figure 10**.

**Figure 8:**
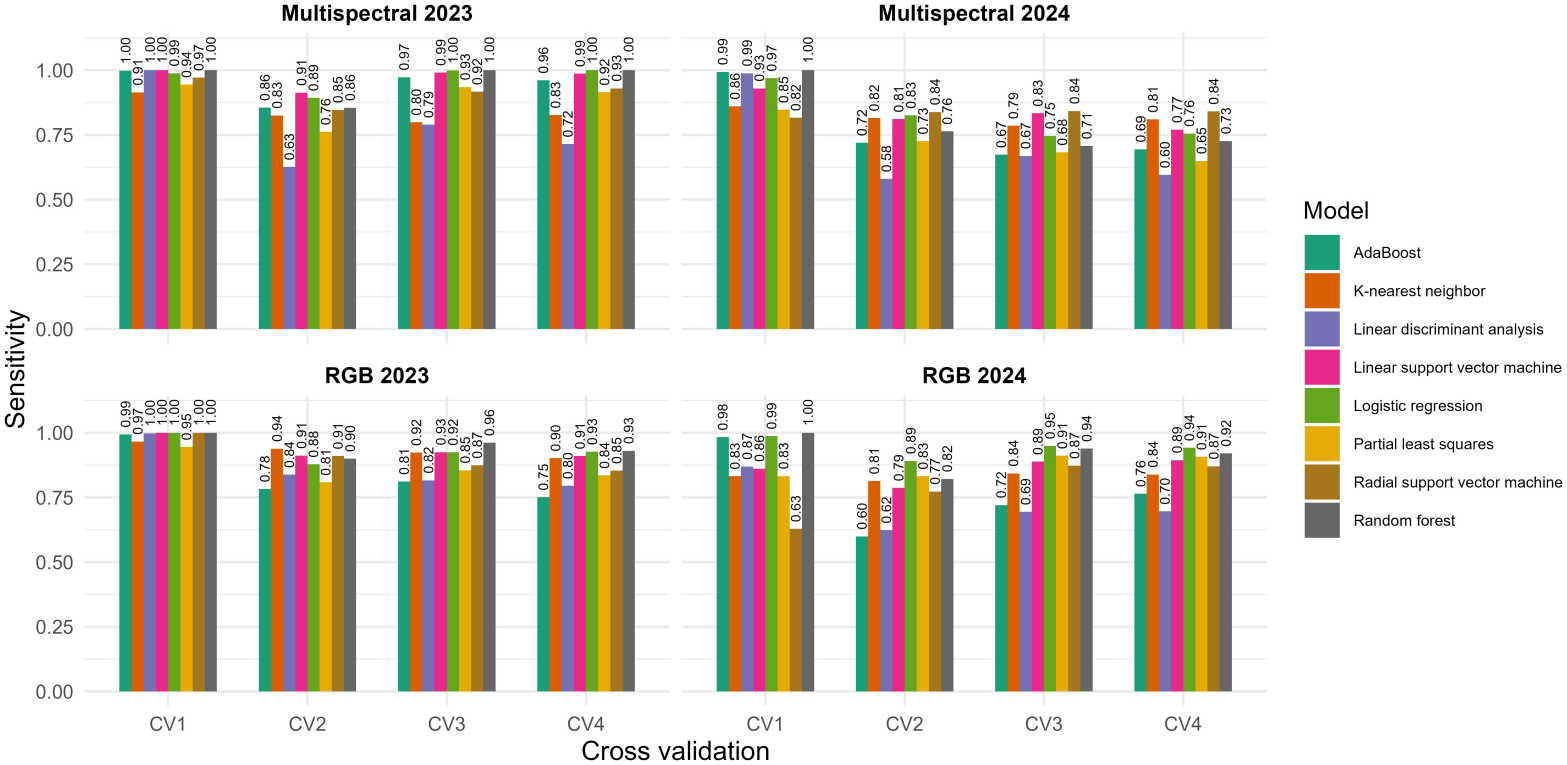
Sensitivity of drought tolerance classification (Y axis) for each combination of sensor and year, across the four cross-validation schemes (X axis) and the eight machine learning models tested. CV1 and CV2 represent the classification of tested and untested genotypes in the observed environment (drought), respectively. CV3 and CV4 represent the classification of tested and untested genotypes in the unobserved environment (irrigated), respectively.

Sensitivity (the rate of genotypes accurately classified as tolerant among all truly tolerant genotypes) varied more across the cross-validation scenarios, but the difference relative to CV1 decreased (**Figure 8**). The models varied significantly across cross-validation scenarios and sensor-year combinations. In CV1, the differences were notable in 2024, where the models AdaBoost, logistic regression, and random forest outperformed the others (sensitivity > 0.9) for the RGB sensor. For the multispectral sensor, these models, along with linear discriminant analysis and linear support vector machine, showed high performance (sensitivity > 0.9).

In CV2, models such as AdaBoost, linear discriminant analysis, and partial least squares did not consistently maintain high sensitivities across all situations. The other models within CV2 regularly ranked among the best. In CV3 and CV4, a pattern emerges where linear discriminant analysis was the lowest-ranked model for the multispectral sensor in both years. Conversely, for the RGB sensor, this model and AdaBoost were the lowest-ranked in both years. The remaining models performed more consistently across years and sensors in the specified cross-validation scenarios. The overall comparison between sensors was opposite in the two years. In 2023, the difference was minimal at 0.88%, while in 2024, the multispectral sensor showed 5.66% lower sensitivity than the RGB sensor. The sensitivities from training the models on irrigated trials are shown in **Supplementary Figure 11**.

**Figure 9:**
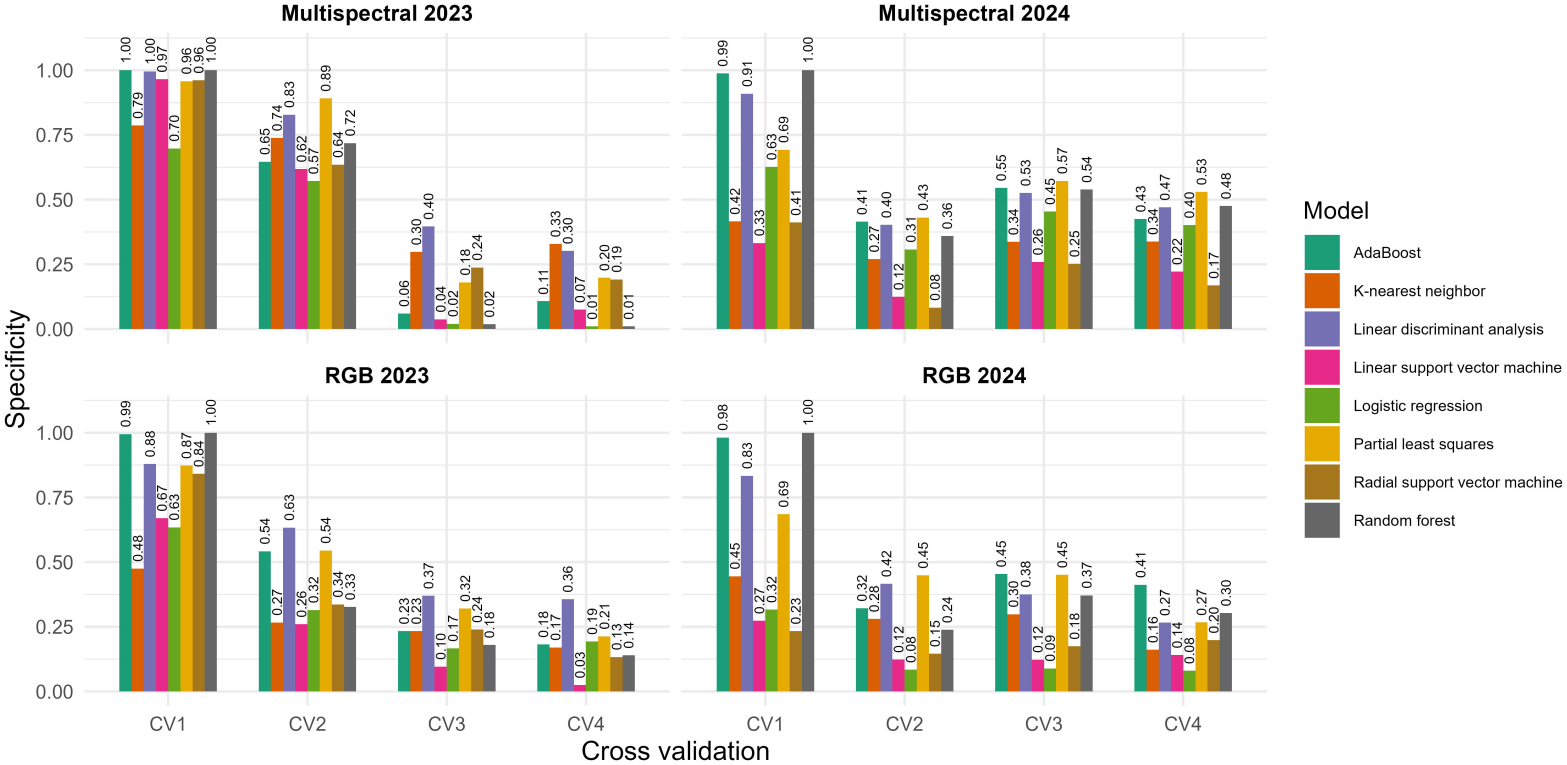
Specificity of drought tolerance classification (Y axis) for each combination of sensor and year, along with the four cross-validation schemes (X axis) and the eight machine learning models examined. CV1 and CV2 represent the classification of tested and untested genotypes in the observed environment (drought), respectively. CV3 and CV4 represent the classification of tested and untested genotypes in the unobserved environment (irrigated), respectively.

Specificity (the rate of genotypes correctly classified as susceptible among all truly susceptible genotypes) was the most discriminative metric for assessing classification efficiency across sensors and years (**Figure 9**). The differences between CV1 and other cross-validation scenarios were significant. In 2024, for CV1, three models stood out for both sensors: AdaBoost, linear discriminant analysis, and random forest (specificity > 0.8). In 2023, the largest differences were observed with the RGB sensor, and these three models also showed higher values. For CV2 in 2023, linear discriminant analysis and partial least squares were the best models (specificity > 0.8). For this same cross-validation scenario and year, but with the RGB sensor, these two models, along with AdaBoost, performed well (specificity > 0.5). These two models were also among the best for CV2 in 2024. Specifically, for the multispectral sensor, AdaBoost stood out alongside them.

For CV3 and CV4, linear discriminant analysis was among the top-performing models for both sensors in 2023. In 2024, AdaBoost, linear discriminant analysis, partial least squares, and random forest were prominent for the multispectral sensor. In that year, for the RGB sensor and CV3 scenario, AdaBoost and partial least squares performed the best, whereas in the CV4 scenario, AdaBoost stood out among the other models (specificity > 0.4). The largest difference in specificity rates between the two sensors was observed in this study. On average, the multispectral sensor achieved 20.64% and 29.88% higher specificity than the RGB sensor in 2023 and 2024, respectively. The specificities for models trained on irrigated trials are shown in **Supplementary Figure 12**.

**Figure 10:**
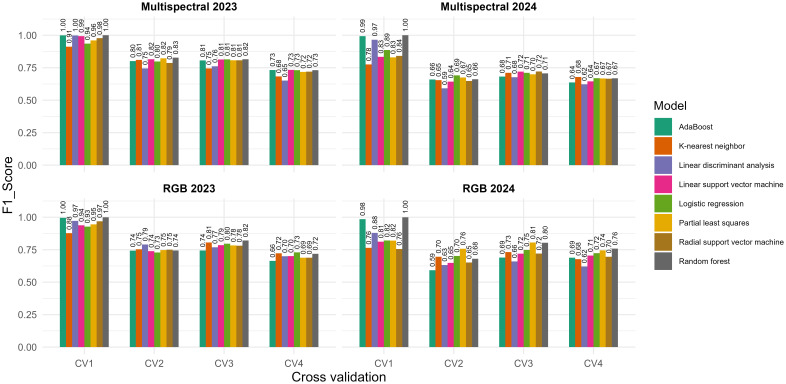
F1-score for drought tolerance classification (Y axis) across each combination of sensor and year, displayed along the four cross-validation schemes (X axis) and the eight machine learning models examined. CV1 and CV2 represent the classification of tested and untested genotypes in the observed environment (drought), respectively. CV3 and CV4 represent the classification of tested and untested genotypes in the unobserved environment (irrigated), respectively.

F1-score (a balance between sensitivity and precision) was uniformly high in most classification scenarios (**Figure 10**). For CV1, the differences became more apparent in 2024, where the models AdaBoost, linear discriminant analysis, and random forest stood out for both sensors. For CV2, CV3, and CV4, the models varied slightly, except for the 2024 RGB sensor, where small differences (>0.1) were observed. The differences between the sensors were also small. The multispectral sensor outperformed RGB by 2.91% in 2023, but in 2024, its advantage decreased to 2.02%. The F1 Scores for training the models on irrigated trials are shown in **Supplementary Figure 13**.

### 3.4 Variable importance

The multispectral sensor outperformed the RGB sensor on most classification-efficiency metrics in both years. Therefore, this sensor was used to investigate the relative importance of predictors, which included a combination of vegetation indices and the date of flight. Among the eight machine learning models, AdaBoost and linear discriminant analysis were the most consistent across the various classification scenarios studied. The only exception was regarding sensitivity, where these two models were not among the top-ranking models in some cross-validation schemes.

**Figure 11:**
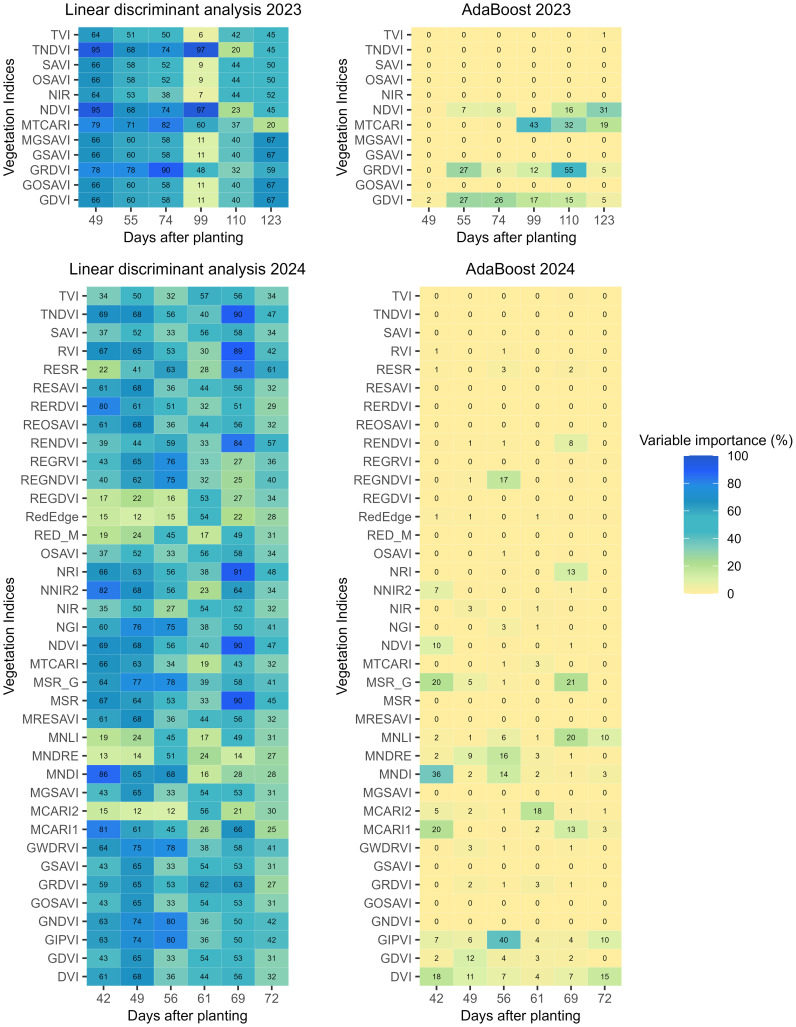
Average relative importance of predictors for the best-performing models, AdaBoost and linear discriminant analysis, for the multispectral sensor in both years. The predictors are shown as combinations of vegetation indices (on the Y-axis) and flight date (on the X-axis).

In linear discriminant analysis, the vegetation indices GRDVI, MTCARI, NDVI, and TNDVI were the most important for classifying genotypes by drought tolerance in 2023 (**Figure 11**). In 2024, the indices MNDI, MSR, NDVI, NRI, RENDVI, RESR, RVI, and TNDVI stood out among the 38 indices used. Notably, all these indices include the NIR band, except RESR and RENDVI. Regarding the most promising dates for flying, they were spread across the 2023 crop cycle, with key flights during the vegetative, flowering, and grain-filling stages that affected model performance. In contrast, in 2024, such dates were mainly concentrated during the flowering stage rather than the vegetative stage.

The AdaBoost model was primarily based on the indices GDVI, GRDVI, MTCARI, and NDVI in 2023. So, this year, three of the four most important indices for each selected model are the same (GRDVI, MTCARI, and NDVI). In 2024, the indices DVI, GIPVI, MCARI1, MCARI2, MNDI, MNDRE, MNLI, MSR_G, and REGNDVI were the most important for AdaBoost performance. Again, all of these indices share the NIR band, except the REGNDVI index. Regarding the best date for flying, the AdaBoost model identified influential flights across all crop stages for both years.

## 4 Discussion

### 4.1 Field trials and drought tolerance classification

Integrating multiple traits to evaluate drought stress tolerance offers advantages that are often overlooked when only final grain yield is considered. Some traits may have higher heritability, be quicker and more cost-effective to assess, and allow for earlier evaluation during the crop cycle (Singh-Bakala et al., 2025; Wen et al., 2023). It has already been reported that spectral traits linked to morpho-physiological adaptations under drought serve as strong markers and proxies for drought resilience (Singh-Bakala et al., 2025). These features together can create a more reliable and robust classification system. MFVD serves as an example of a multi-trait index that thoroughly assesses performance during drought conditions.

Stable traits across years and varying environmental conditions deserve special attention, as their phenotypic expressions better reflect genotypic effects rather than environmental fluctuations. As we observed, plant height and hundred-grain weight were also stable traits across years and stress conditions in Singh et al. (2018) with bread wheat. Additionally, using MFVD to classify drought tolerance in cotton based on 19 traits, Sun et al. (2021) identified five traits as key indicators of drought tolerance. This subset of five traits could classify cotton varieties with good agreement compared to the full set of 19 traits, including plant height. The measurement of this trait can be easily automated through aerial imagery phenotyping and is related to induced plant adaptations to cope with drought (Su et al., 2019). Therefore, it should be considered in future studies on plant stress tolerance.

### 4.2 Vegetation indices

The vegetation indices showed strong sensitivity to environmental changes over the two years. This new category of trait, based on the spectral reflectance of plant tissues, accurately measures the induced changes (Alves et al., 2024; Asaari et al., 2019; Wang et al., 2021). Supporting our findings, Abdollahnejad and Panagiotidis (2020) also noted significant differences between summer and spring seasons for some multispectral indices across four tree species classes. RGB and multispectral sensors could become valuable tools in plant research, as they enable automation, are non-invasive, and can provide near real-time information on plant physiological status.

High-throughput phenotyping using UAS allows us to monitor plant responses over time, helping to explain much of the variation. The flight effect was dominant across all years, sensors, and trials, except for the multispectral sensor in 2024, when flights were limited to vegetative and early flowering stages. Therefore, when making point evaluations, which is common in field trials due to cost and labor constraints, an important source of variation has been overlooked. The correlations between vegetation indices and agronomic traits can vary significantly over time as the crop develops, and also among different environments for the same index (Alves et al., 2024). Multi-modal data (RGB, hyperspectral, 3D, and temporal) in studies of plant stress has shown the best classification performance when time is included as an input feature, outperforming all individual modalities (Khanna et al., 2019). Even time-invariant deep learning methods, such as convolutional neural networks, benefited from temporal features when classifying maize under water stress (Nampally et al., 2023).

This work demonstrated the feasibility of using vegetation indices to assess the drought tolerance of maize hybrids over two years. Therefore, high-throughput phenotyping with images can complement and reduce reliance on traditional multi-trait field evaluations, helping plant research teams save time and resources.

### 4.3 Drought tolerance classification from spectral information

The nature of training data significantly influences drought tolerance classification, and drought trials provide the most reliable data for training machine learning models. In our research, this data was used as input variables to predict genotypes under drought conditions (CV1 and CV2) as well as irrigated conditions (CV3 and CV4). Li et al. (2022) also noted improved results when training the model with data from drought trials and employing three multispectral vegetation indices (NDVI, NDRE, RDVI) to forecast fresh and dry biomass in sorghum. Consistent with our findings, Ryckewaert et al. (2021) identified a very similar drought tolerance classification of maize hybrids during moderate and severe drought stress years, using spectral data from the more drought-stressed year to train the model.

Specificity (true negative rate) is rarely reported in the literature, although it was the evaluation metric most affected by the nature of the training data and type of sensor in our study. Additionally, depending on the application, the cost of misclassifying the negative class (false positives) can be high, as in studies of human diseases and fraud detection. In our case, the positive class (tolerant) was the main focus, and misclassifying it would be more harmful. However, understanding how well the negative class (susceptible) is predicted provides an extra benefit, helping other groups adapt our pipeline to their specific needs. When there is class imbalance, as in our study, it is advisable to evaluate multiple metrics to select the best model and understand the behavior of the minority class. The dominant class can skew some metrics, such as accuracy (Naik et al., 2017). An alternative to deal with it is the balanced accuracy, that ensures each class contributing equally to the final score. In the case of binary classification, balanced accuracy is the mean of sensitivity and specificity. In **Supplementary Figure 14** we can see that balanced accuracy in our study did not differ so drastically from traditional accuracy (**Figure 6**). The values are lower, as expected, but the same pattern of sensors and models within each cross-validation scenario remains very similar. In the case where we can assign different costs for the misclassifications, that is, false positives and false negatives, another alternative for classification is by cost sensitive learning. In this approach a matrix of misclassification costs is used for training the models, whose algorithms try to minimize the total misclassification cost instead of errors (Araf et al., 2024).

For drought-tolerance classification, investing in a more expensive sensor can be justified by the generally better performance of multispectral sensors compared with RGB sensors. The literature supports our findings with other species. Abdollahnejad and Panagiotidis (2020) reported high overall accuracy (>0.80) for classifying health states (healthy, infected, and dead) of trees using multispectral vegetation indices and a support vector machine. Promising results in classifying soybean varieties for drought stress, based on their wilting symptoms observed from aerial imagery, were reported by Jones et al. (2024). Multispectral vegetation indices performed best in their study, achieving an overall accuracy of 0.71 for monitoring and 0.60 for early detection of soybean wilt. Li et al. (2022) found that evaluating multimodal data to predict sorghum’s fresh and dry biomass under both well-watered and water-stressed conditions yielded the best results using multispectral sensors compared with RGB and thermal sensors. Additionally, multispectral traits had the highest classification success—compared to morphological and chlorophyll fluorescence traits—in detecting nutritional stress (N, P, K, Mg, and Fe) in common beans (Lazarević et al., 2022).

Our work provided a comprehensive assessment of machine learning models for classifying genotypes under stressful conditions across various real-world scenarios. These models addressed a broad spectrum of machine learning assumptions and algorithms (Gill et al., 2022). We confirmed that there is no single best model for all situations, as we studied different sensors, environments, and prediction scenarios. However, some models are more consistent than others and show promise for future research. If a researcher has a specific prediction need, our results can guide their study, as they cover a wide range of conditions commonly encountered in breeding programs. Our findings indicate that model choice has significant implications for drought tolerance classification. This is supported by other studies involving similar classification tasks for this type of stress (Duarte-Carvalajino et al., 2021; Guo et al., 2017).

### 4.4 Variable importance

The NIR band appears to underpin the superiority of multispectral sensors, as it was present in most of the key vegetation indices used for drought tolerance classification, including the two best-performing models. It has already been reported that including NIR and RedEdge bands improves accuracy, precision, and sensitivity compared to using only RGB bands for maize classification under stress (Nampally et al., 2023). The NIR band also influences the vegetation indices used to classify cotton cultivars under four levels of water stress across three yield groups (high, medium, and low) (Gu et al., 2024). These authors also noted high correlation coefficients between four NIR-based vegetation indices (EVI, GNDVI, NDRE, NDVI) measured during mid- and late-season flights and cotton yield. On the other hand, for early stress detection in soybeans, Jones et al. (2024) identified the RedEdge as the most informative band, while the NIR band also contributes to stress monitoring.

In line with the property of the NIR band to reflect the water status of tissues, Lad et al. (2023) found this band (820–860 nm) to be the most effective for distinguishing four drought groups, and also for predicting foliar moisture content using the NDVI index in two conifer species. The NDVI vegetation index deserves special attention in studies of water stress, as our results highlight it as the most important predictor among the top ones in both models and years. This finding is supported by Zhang et al. (2021), in which NDVI was the vegetation index that best explained maize’s response to water stress, showing the highest correlations with stomatal conductance and leaf area index.

The environment plays a significant role in the most important vegetation indices for prediction, as these indices vary widely between the two years within each model. Dissecting the genetics of NDVI dynamics over time, Wang et al. (2021) found significant changes in the additive effect sizes and directions of SNPs, suggesting that gene-environment interactions are important for NDVI expression. Therefore, exploring high-throughput phenotyping using images offers greater sensitivity to environmental fluctuations in plant responses (Alves et al., 2024). In line with this, our results showed that drought tolerance classification depends on spectral information from all crop stages, rather than point evaluations. Therefore, it is recommended to distribute the flights/evaluations throughout the entire crop cycle, as each includes vegetation indices that determine model performance.

We believe that better results of plant’s response classification can be obtained with larger data sets, broader environmental sampling, deeper drought assessment by deterministic traits and more robust options for analysis. Further studies can confirm the robustness of our drought tolerance classification pipeline, as our study addressed a single location, reduced sampling of genotypes and missing data of the multispectral sensor in one year.

## 5 Conclusion

Spectral information derived from UAS imagery, particularly vegetation indices, is a valuable tool for plant researchers to assess drought tolerance in the field. This trait can support a data-driven approach that enables automation and improves resource use efficiency. Vegetation indices should be measured throughout the crop cycle, as key predictors are present at all phenological stages. The NIR band plays a significant role in classifying drought tolerance, making multispectral sensors the preferred option for this purpose.

Overall, UAS-based phenotyping shows strong potential to support drought-tolerance classification in breeding scenarios, although further validation is needed to improve generalization. These findings highlight the potential of integrating UAS-based phenotyping into breeding pipelines to develop climate-resilient crops.

## Data Availability

A dataset containing an example orthomosaic (TIF format) and its corresponding polygons from a shapefile (from QGIS software) used to clip the plots and calculate vegetation indices was provided in the folder “Extracting vegetation indices.” The membership function values at the trait and genotype levels (Uij and Ui), along with the corresponding drought tolerance classifications of genotypes from field traits, to feed the machine learning models for all cross-validation scenarios, were provided as examples in the folder “Spectral drought tolerance classification.” All the codes and datasets described, along with supporting information for users, are available in the article/**Supplementary Material**.
