# Supplementary material for: Drought tolerance classification using unmanned aerial systems based on RGB and multispectral data
Source: Front Plant Sci. 2026 Jun 29;17:1853372. doi: 10.3389/fpls.2026.1853372 (PMC13357819; doi:10.3389/fpls.2026.1853372)
Supplement: Supplementary file 1 [file Table1.docx]

**Drought tolerance classification using unmanned aerial systems based on RGB and multispectral data**

Supplementary Table S1: List of the genotypes used in this study.

| **Hybrid** | **ID** |
| --- | --- |
| 06GC90_III_6f.1 x 488 | G1 |
| GC_140 x 360 | G2 |
| 25GC66_VII_2 x 488 | G3 |
| 34GC66_III_20 x 488 | G4 |
| 25GC66_VII_2 x 360 | G5 |
| GCUCP x 360 | G6 |
| 25GC66_VII_1 x 360 | G7 |
| 25GC66_VII_1 x 488 | G8 |
| GCUCP x 488 | G9 |
| GC_A21 x 360 | G10 |
| 25GC66_I_2 x 360 | G11 |
| GC_140 x 488 | G12 |
| 06GC90_III_6f.1 x 360 | G13 |
| AG8088 | G14 |
| 34GC66_III_20 x 360 | G15 |
| GC_A14 x 488 | G16 |
| B104 x 488 | G17 |
| 25GC66_I_2 x 488 | G18 |
| B104 x 360 | G19 |
| GC_A14 x 360 | G20 |
| GC_A21 x 488 | G21 |
| HiII x 360 | G22 |
| HiII x 488 | G23 |
| AG8606 | G24 |
| GC_139 x 360 | G25 |
| GC_139 x 488 | G26 |
| GC_07 x 360 | G27 |
| GC_07 x 488 | G28 |

Supplementary Table S2: RGB vegetation indices used and their expressions along with the references

| **RGB Vegetation index** | **Expression*** | **References** |
| --- | --- | --- |
| Blue chromatic coordinate index (BCC) | $\frac{B}{R+G+B}$ | (Woebbecke et al. 1995) |
| Blue green pigment index (BGI) | $\frac{B}{G}$ | (Zarco-Tejada et al. 2005) |
| Brightness index (BI) | $sqrt(\frac{R^{2}+G^{2}+B^{2}}{3})$ | (Richardson and Wiegand 1977) |
| Color index of vegetation extraction (CIVE) | $0.441R-0.811G+$  $0.385B+18.78745$ | (Kataoka et al. 2003) |
| Combined indices 1 (COM1) | $EXG+CIVE+EXGR+VEG$ | (Guijarro et al. 2011) |
| Combined indices 2 (COM2) | $0.36EXG+0.47CIVE+0.17VEG$ | (Guerrero et al. 2012) |
| Additional blue index (EBI) | $\frac{B-G}{B-R}$ | (Golzarian and Frick 2011) |
| Additional green index (EGI) | $\frac{G-R}{R-B}$ | (Golzarian and Frick 2011) |
| Green-red index (ERI) | $\frac{R-G}{R-B}$ | (Golzarian and Frick 2011) |
| Excessive green (EXG) | $2G-R-B$ | (Woebbecke et al. 1995) |
| Normalized Excess green index (EXG2) | $\frac{2G-R-B}{G+R+B}$ | (Woebbecke et al. 1995) |
| Excess green minus excess red index (EXGR) | $3G-2.4R-B$ | (Meyer and Neto 2008) |
| Excessive red (EXR) | $1.4R-G$ | (Meyer et al. 1999) |
| Green minus blue index (G_B) | $G-B$ | (Woebbecke et al. 1995) |
| Green minus red index (G_R) | $G-R$ | (Woebbecke et al. 1995) |
| Green blue simple ratio index (GB) | $\frac{G}{B}$ | (Woebbecke et al. 1995) |
| Green red simple ratio index (GR) | $\frac{G}{R}$ | (Woebbecke et al. 1995) |
| Green chromatic coordinate index (GCC) | $\frac{G}{R+G+B}$ | (Woebbecke et al. 1995) |
| Green leaf index (GLI) | $\frac{2G-R-B}{2G+R+B}$ | (Louhaichi et al. 2001) |
| Modified excess green index (MEXG) | $1.262G-0.884R$  $-0.311B$ | (Burgos-Artizzu et al. 2011) |
| Modified green red index (MGVRI) | $\frac{G^{2}-R^{2}}{G^{2}+R^{2}}$ | (Bendig et al. 2015) |
| Normalized difference index (NDI) | $128*[\left( \frac{\left( G-R \right)}{\left( G+R \right)} \right)+1]$ | (Meyer and Neto 2008) |
| Normalized difference red  blue index (NDRBI) | $\frac{R-B}{R+B}$ | (Golzarian and Frick 2011) |
| Normalized green-blue difference index (NGBDI) | $\frac{G-B}{G+B}$ | (Hunt et al. 2005) |
| Normalized green red difference index (NGRDI) | $\frac{G-R}{G+R}$ | (Tucker 1979) |
| Red minus blue index (R_B) | $R-B$ | (Woebbecke et al. 1995) |
| Red blue simple ratio index (RB) | $\frac{R}{B}$ | (Woebbecke et al. 1995) |
| Red chromatic coordinate index (RCC) | $\frac{R}{R+G+B}$ | (Woebbecke et al. 1995) |
| Red green blue index (RGBVI) | $\frac{G^{2}-R*B}{G^{2}+R*B}$ | (Bendig et al. 2015) |
| Triangular greenery index (TGI) | $G-(0.39R-0.69B)$ | (Hunt et al. 2011) |
| Visible atmospherically resistant index (VARI) | $\frac{G-R}{G+R-B}$ | (Gitelson et al. 2002) |
| Vegetativen (VEG) | $\frac{G}{R^{0.667}*B^{0.334}}$ | (Hague et al. 2006) |

* R, G and B means red, green and blue bands

Supplementary Table S3: Multispectral vegetation indices used and their expressions along with the references

| **Multispectral Vegetation index** | **Expression*** | **References** |
| --- | --- | --- |
| Modified chlorophyll absorption in reflectance index 1(MCARI1) | $\left[ \left( \mathrm{NIR}- \mathrm{RE} \right)- 0.2 *\left( \mathrm{NIR}- G \right) \right]*\left( \frac{\mathrm{NIR}}{\mathrm{RE}} \right)$ | (Daughtry et al. 2000) |
| Modified chlorophyll absorption in reflectance index 2(MCARI2) | $\frac{1.5\left( NIR-RE \right)-1.3\left( NIR-G \right)}{\sqrt{\left( 2NIR+1 \right)^{2}-\left( 6NIR-5\sqrt{\mathrm{RE}} \right)-0.5}}$ | (Haboudane et al. 2004) |
| Chlorophyll vegetation index-green (CIG) | $\frac{\mathrm{NIR}}{G}-1$ | (Gitelson et al. 2005) |
| Chlorophyll vegetation index-red edge (CIRE) | $\frac{\mathrm{NIR}}{\mathrm{RE}}-1$ | (Gitelson et al. 2005) |
| Chlorophyll vegetation index (CVI) | $\frac{NIR*R}{G^{2}}$ | (Vincini et al. 2008) |
| Difference vegetation index (DVI) | $NIR-RE$ | (Tucker 1979) |
| Green difference vegetation index (GDVI) | $NIR-G$ | (Tucker 1979) |
| Green infrared percentage vegetation index (GIPVI) | $\frac{\mathrm{NIR}}{NIR+G}$ | (Crippen 1990) |
| Green normalized difference vegetation index (GNDVI) | $\frac{NIR-G}{NIR+G}$ | (Gitelson et al. 1996) |
| Green optimal soil adjusted vegetation index (GOSAVI) | $\frac{\left( 1+0.16 \right)\left( NIR-G \right)}{NIR+G+0.16}$ | (Rondeaux et al. 1996) |
| Green re-normalized different vegetation index (GRDVI) | $\frac{NIR-G}{\mathrm{sqrt}\left( NIR+G \right)}$ | (Roujean and Breon 1995) |
| Green ratio vegetation index (GRVI) | $\frac{\mathrm{NIR}}{G}$ | (Buschmann and Nagel 1993) |
| Green soil adjusted vegetation index (GSAVI) | $1.5\left( \frac{NIR-G}{NIR+G+0.5} \right)$ | (Sripada et al. 2006) |
| Green wide dynamic range vegetation index (GWDRVI) | $\frac{0.12NIR-G}{0.12NIR+G}$ | (Gitelson 2004) |
| Modified double difference index (MDD) | $(NIR-RE)-(RE-G)$ | (Le Maire et al. 2004) |
| Modified GSAVI (MGSAVI) | $0.5[2NIR + 1 - sqrt((2NIR + 1)^{2}- 8(NIR - G))]$ | (Qi et al. 1994) |
| Modified normalized difference index (MNDI) | $(NIR-RE)/(NIR-G)$ | (Datt 1999) |
| Modified normalized difference red edge (MNDRE) | $\frac{\left[ NIR-\left( RE-2G \right) \right]}{\left[ NIR+\left( RE-2G \right) \right]}$ | (Wang et al. 2012) |
| Modified RESAVI (MRESAVI) | $0.5 [2NIR + 1 - sqrt((2NIR + 1)^{2}- 8(NIR - RE))]$ | (Qi et al. 1994) |
| Modified RETVI (MRETVI) | 1.2[1.2(NIR − G) − 2.5(RE − G)] | (Haboudane et al. 2004) |
| Modified simple ratio (MSR) | $\frac{\left( \frac{\mathrm{NIR}}{R}-1 \right)}{\sqrt{\left( \frac{\mathrm{NIR}}{R}+1 \right)}}$ | (Chen 1996) |
| Modified green simple  ratio (MSR_G) | $\frac{\left( \frac{\mathrm{NIR}}{G}-1 \right)}{\sqrt{\left( \frac{\mathrm{NIR}}{G}+1 \right)}}$ | (Chen 1996) |
| Modified green simple  ratio (MSR_RE) | $\frac{\left( \frac{\mathrm{NIR}}{\mathrm{RE}}-1 \right)}{\sqrt{\left( \frac{\mathrm{NIR}}{\mathrm{RE}}+1 \right)}}$ | (Chen 1996) |
| Modified transformed  CARI (MTCARI) | $3\left[ \left( \mathrm{NIR}- \mathrm{RE} \right)- 0.2\left( \mathrm{NIR}- G \right)\left( \frac{\mathrm{NIR}}{\mathrm{RE}} \right) \right]$ | (Haboudane et al. 2002) |
| Normalized difference  red edge (NDRE) | $\frac{\mathrm{NIR}- \mathrm{RE}}{NIR+ \mathrm{RE}}$ | (Barnes et al. 2000) |
| Normalized difference vegetation index (NDVI) | $\frac{\mathrm{NIR}- R}{NIR+ R}$ | (Tucker 1979) |
| Normalized NIR index (NNIR) | $\frac{\mathrm{NIR}}{NIR+RE+ G}$ | (Sripada et al. 2006) |
| Normalized red edge index (NREI) | $\frac{\mathrm{RE}}{NIR+RE+ G}$ | (Sripada et al. 2006) |
| Normalized green index (NGI) | $\frac{G}{NIR+RE+ G}$ | (Sripada et al. 2006) |
| Optimized soil-adjusted vegetation index (OSAVI) | $\frac{NIR-R}{NIR+R+0.16}$ | (Rondeaux et al. 1996) |
| Plant senescence reflectance index (PSRI) | $\frac{R-G}{\mathrm{RE}}$ | (Merzlyak et al. 1999) |
| Red edge green difference vegetation index (REGDVI) | $RE-G$ | (Tucker 1979) |
| Red edge GNDVI (REGNDVI) | $\frac{RE-G}{RE+G}$ | (Gitelson et al. 1996) |
| Red edge green ratio vegetation index (REGRVI) | $\frac{\mathrm{RE}}{G}$ | (Cao et al. 2013) |
| Red edge optimal soil adjusted vegetation index (REOSAVI) | $\frac{\left( 1+0.16 \right)\left( NIR-RE \right)}{NIR+RE+0.16}$ | (Rondeaux et al. 1996) |
| Renormalized difference vegetation index (RERDVI) | $\frac{NIR-RE}{\sqrt{NIR+RE}}$ | (Roujean and Breon 1995) |
| Red edge soil adjusted vegetation index (RESAVI) | $1.5\left[ \frac{NIR- RE}{NIR + RE + 0.5} \right]$ | (Sripada et al. 2006) |
| Red edge transformed vegetation index (RETVI) | 0.5[120(NIR − G) − 200(RE − G)] | (Broge and Leblanc 2001) |
| Red edge wide dynamic range vegetation index (REWDRVI) | $\frac{0.12NIR-RE}{0.12NIR+RE}$ | (Gitelson 2004) |
| Ratio vegetation index (RVI) | $\frac{\mathrm{NIR}}{R}$ | (Jordan 1969) |
| Soil-adjusted vegetation index (SAVI) | $\frac{1.5\left( NIR-R \right)}{NIR+R+0.5}$ | (Huete 1988) |
| Triangular vegetation index (TVI) | 0.5[120(NIR − G) − 200(R − G)] | (Broge and Leblanc 2001) |
| Optimized vegetation  index 1 (VIopt1) | 100(lnNIR – lnRE) | (Jasper et al. 2009) |
| Transformed Normalized Vegetation Index (TNDVI) | $\mathrm{sqrt}\left( \frac{NIR-R}{NIR+R}+0.5 \right)$ | (Sandham and Zietsman 1997) |
| Modified Nonlinear Index (MNLI) | $\frac{1.5\left( NIR^{2}-R \right)}{NIR^{2}+R+0.5}$ | (Gong et al. 2003) |
| Red Edge Simple Ratio (RESR) | $\frac{RE}{R}$ | (Erdle et al. 2011) |
| Red edge normalized difference vegetation index (RENDVI) | $\frac{RE-R}{RE+R}$ | (Elsayed et al. 2015) |
| Normalized NIR index2 (NNIR2) | $\frac{\mathrm{NIR}}{NIR+RE+ R}$ | (Sripada et al. 2006) |
| Normalized red edge index2 (NREI2) | $\frac{\mathrm{RE}}{NIR+RE+ R}$ | (Sripada et al. 2006) |
| Normalized red index (NRI) | $\frac{R}{NIR+RE+ R}$ | (Sripada et al. 2006) |
| Green single band (Green_M) | 560 ±16 nm** | - |
| Red single band (Red_M) | 650 ±16 nm** | - |
| Red-Edge single band (RedEdge) | 730 ±16 nm** | - |
| Near infra-red single band (NIR) | 860 ±26 nm** | - |

* R, G, RE and NIR means red, green, red-edge and near infra-red bands

** Wavelength band of the multispectral sensor

Supplementary Table S4: Membership function value to drought at trait (U_ij_) and genotype level (U_i_), jointly with the classification of the genotypes by k-means clustering. The values of U_ij_ are presented within each year. The traits are: plant height (PH), ear height (EH), anthesis silking interval (ASI), hundred grain weight (HGW), grain yield (GY), ear length (EL) and ear diameter (ED).

| **Genotype** | **Year** | | | | | | | | | | | | **MFVD (U_i_)** | **Group** |
| --- | --- | --- | --- | --- | --- | --- | --- | --- | --- | --- | --- | --- | --- | --- |
|  | **2023** | | | | | | |  | **2024** | | | |  |  |
|  | **PH** | **EH** | **ASI** | **HGW** | **GY** | **EL** | **ED** |  | **PH** | **ASI** | **HGW** | **GY** |  |  |
| G1 | 0.6154 | 0.2028 | 0.6296 | 0.7545 | 0.8006 | 1.0000 | 1.0000 |  | 0.3184 | 1.0000 | 0.5020 | 0.7462 | 0.6881 | Tolerant |
| G2 | 0.2127 | 1.0000 | 1.0000 | 0.2069 | 0.2921 | 0.0000 | 0.0000 |  | - | 0.0000 | 0.3041 | 1.0000 | 0.4016 | Susceptible |
| G3 | 0.6571 | 0.6955 | 0.0000 | 0.5499 | 0.7553 | 0.7927 | 0.5866 |  | 0.7260 | 0.0000 | 0.7797 | 0.6993 | 0.5675 | Susceptible |
| G4 | 0.8215 | 1.0000 | 0.7130 | 0.3075 | 0.4271 | 0.2578 | 0.5427 |  | 0.7220 | 0.0000 | 0.3152 | 0.2247 | 0.4847 | Susceptible |
| G5 | 0.2978 | 0.3880 | 0.9267 | 0.4491 | 0.8359 | 0.7872 | 0.7021 |  | - | 0.7077 | 0.1999 | 0.6783 | 0.5973 | Tolerant |
| G6 | 0.0000 | 0.0000 | 0.5337 | 0.0000 | 0.3478 | 0.0083 | 0.2316 |  | - | 1.0000 | 0.0000 | 0.4081 | 0.2529 | Tolerant |
| G7 | 0.2739 | 0.1038 | 0.7625 | 0.3344 | 0.3996 | 0.4780 | 0.3319 |  | 0.4851 | 0.8738 | 0.3199 | 0.0000 | 0.3966 | Tolerant |
| G8 | 0.6332 | 0.4990 | 0.9915 | 0.6310 | 0.4490 | 0.6166 | 0.3700 |  | 0.8196 | 0.5551 | 0.1386 | 0.4008 | 0.5549 | Tolerant |
| G9 | 0.3712 | 0.2921 | 0.4149 | 0.5011 | 0.2265 | 0.6677 | 0.5788 |  | 0.5108 | 1.0000 | 0.1207 | 0.1862 | 0.4427 | Tolerant |
| G10 | 0.3974 | 0.2184 | 0.7103 | 1.0000 | 0.8947 | 1.0000 | 0.6672 |  | 0.3176 | 0.7306 | 0.7811 | 0.4355 | 0.6502 | Tolerant |
| G11 | 1.0000 | 0.6696 | 0.8815 | 0.4343 | 0.4912 | 0.5094 | 0.2592 |  | 0.0000 | 1.0000 | 0.5947 | 0.7183 | 0.5962 | Tolerant |
| G12 | 0.6328 | 0.9076 | 1.0000 | 0.9042 | 0.0000 | 0.7655 | 0.8512 |  | 0.7979 | 0.4953 | 1.0000 | 1.0000 | 0.7595 | Tolerant |
| G13 | 0.8789 | 0.5950 | 1.0000 | 0.5843 | 1.0000 | 1.0000 | 0.6483 |  | 0.7008 | 1.0000 | 0.7644 | 1.0000 | 0.8338 | Tolerant |
| G14 | 0.5414 | 0.3417 | 0.5408 | 0.7313 | 0.7174 | 0.3915 | 0.4771 |  | 0.8149 | 0.0000 | 0.8410 | 0.6278 | 0.5477 | Susceptible |
| G15 | 0.3928 | 0.2519 | 0.8534 | 0.6717 | 0.6407 | 0.9585 | 0.7423 |  | 0.7806 | 1.0000 | 0.2995 | 0.6049 | 0.6542 | Tolerant |
| G16 | 0.6214 | 0.7328 | 0.9003 | 0.7762 | 0.4440 | 0.7805 | 0.5425 |  | 0.6568 | 0.0043 | 0.3013 | 0.0214 | 0.5256 | Susceptible |
| G17 | 0.9735 | 0.4164 | 0.8788 | 0.9099 | 0.7724 | 1.0000 | 0.8340 |  | 0.3984 | 0.0000 | 0.6670 | 0.7160 | 0.6879 | Tolerant |
| G18 | 0.4542 | 0.3092 | 1.0000 | 0.7885 | 0.2507 | 0.7964 | 0.9132 |  | 0.7015 | 1.0000 | 0.4975 | 0.7298 | 0.6765 | Tolerant |
| G19 | 0.2747 | 0.5705 | 0.6261 | 0.1799 | 0.3278 | 0.0850 | 0.1858 |  | - | 0.0000 | 0.6830 | 1.0000 | 0.3933 | Susceptible |
| G20 | 0.6114 | 0.3630 | 0.5969 | 0.4700 | 0.5977 | 0.8691 | 0.4371 |  | 0.2790 | 0.7005 | 0.2350 | 0.9296 | 0.5536 | Tolerant |
| G21 | 0.1048 | 0.1956 | 0.7783 | 0.6551 | 0.5320 | 0.2064 | 0.5777 |  | 0.7065 | 0.0000 | 0.4647 | 0.0852 | 0.3915 | Susceptible |
| G22 | - | - | - | - | - | - | - |  | - | 1.0000 | 0.4390 | 1.0000 | 0.8130 | Tolerant |
| G23 | - | - | - | - | - | - | - |  | 0.8511 | 0.4235 | 0.7378 | 1.0000 | 0.7531 | Tolerant |
| G24 | - | - | - | - | - | - | - |  | 1.0000 | 0.0000 | 0.8715 | 0.1848 | 0.5141 | Susceptible |
| G25 | - | - | - | - | - | - | - |  | - | 1.0000 | 1.0000 | 1.0000 | 1.0000 | Tolerant |
| G26 | - | - | - | - | - | - | - |  | 0.7566 | 0.2247 | 0.4254 | 0.0322 | 0.3597 | Susceptible |
| G27 | - | - | - | - | - | - | - |  | 0.0324 | 0.4986 | 0.1303 | 0.2554 | 0.2292 | Susceptible |
| G28 | - | - | - | - | - | - | - |  | 0.0871 | 0.8740 | 0.5196 | 0.0246 | 0.3763 | Susceptible |

**References**

Barnes, E. M., Clarke, T. R., Richards, S. E., Colaizzi, P. D., Haberland, J., Kostrzewski, M., ... & Moran, M. S. (2000, July). Coincident detection of crop water stress, nitrogen status and canopy density using ground based multispectral data. In *Proceedings of the fifth international conference on precision agriculture, Bloomington, MN, USA* (Vol. 1619, No. 6).

Bendig, J., Yu, K., Aasen, H., Bolten, A., Bennertz, S., Broscheit, J., ... & Bareth, G. (2015). Combining UAV-based plant height from crop surface models, visible, and near infrared vegetation indices for biomass monitoring in barley. *International Journal of Applied Earth Observation and Geoinformation*, 39, 79-87.

Broge, N. H., & Leblanc, E. (2001). Comparing prediction power and stability of broadband and hyperspectral vegetation indices for estimation of green leaf area index and canopy chlorophyll density. *Remote sensing of environment*, 76(2), 156-172.

Burgos-Artizzu, X. P., Ribeiro, A., Guijarro, M., & Pajares, G. (2011). Real-time image processing for crop/weed discrimination in maize fields. *Computers and Electronics in Agriculture*, 75(2), 337-346.

Buschmann, C., & Nagel, E. (1993). In vivo spectroscopy and internal optics of leaves as basis for remote sensing of vegetation. *International Journal of Remote Sensing*, 14(4), 711-722.

Cao, Q., Miao, Y., Wang, H., Huang, S., Cheng, S., Khosla, R., & Jiang, R. (2013). Non-destructive estimation of rice plant nitrogen status with Crop Circle multispectral active canopy sensor. *Field Crops Research*, 154, 133-144.

Chen, J. M. (1996). Evaluation of vegetation indices and a modified simple ratio for boreal applications. *Canadian Journal of Remote Sensing*, 22(3), 229-242.

Crippen, R. E. (1990). Calculating the vegetation index faster. *Remote sensing of Environment*, 34(1), 71-73.

Datt, B. (1999). Visible/near infrared reflectance and chlorophyll content in Eucalyptus leaves. *International Journal of Remote Sensing*, 20(14), 2741-2759.

Daughtry, C. S., Walthall, C. L., Kim, M. S., De Colstoun, E. B., & McMurtrey Iii, J. E. (2000). Estimating corn leaf chlorophyll concentration from leaf and canopy reflectance. *Remote sensing of Environment*, 74(2), 229-239.

Elsayed, S., Rischbeck, P., & Schmidhalter, U. (2015). Comparing the performance of active and passive reflectance sensors to assess the normalized relative canopy temperature and grain yield of drought-stressed barley cultivars. *Field Crops Research*, 177, 148-160.

Erdle, K., Mistele, B., & Schmidhalter, U. (2011). Comparison of active and passive spectral sensors in discriminating biomass parameters and nitrogen status in wheat cultivars. *Field Crops Research*, 124(1), 74-84.

Gitelson, A. A. (2004). Wide dynamic range vegetation index for remote quantification of biophysical characteristics of vegetation. *Journal of plant physiology*, 161(2), 165-173.

Gitelson, A. A., Viña, A., Ciganda, V., Rundquist, D. C., & Arkebauer, T. J. (2005). Remote estimation of canopy chlorophyll content in crops. *Geophysical research letters*, 32(8).

Gitelson, A. A., Kaufman, Y. J., & Merzlyak, M. N. (1996). Use of a green channel in remote sensing of global vegetation from EOS-MODIS. *Remote sensing of Environment*, 58(3), 289-298.

Gitelson, A. A., Kaufman, Y. J., Stark, R., & Rundquist, D. (2002). Novel algorithms for remote estimation of vegetation fraction. *Remote sensing of Environment*, 80(1), 76-87.

Golzarian, M. R., & Frick, R. A. (2011). Classification of images of wheat, ryegrass and brome grass species at early growth stages using principal component analysis. *Plant methods*, 7, 1-11.

Gong, P., Pu, R., Biging, G. S., & Larrieu, M. R. (2003). Estimation of forest leaf area index using vegetation indices derived from Hyperion hyperspectral data. *IEEE transactions on geoscience and remote sensing*, 41(6), 1355-1362.

Guerrero, J. M., Pajares, G., Montalvo, M., Romeo, J., & Guijarro, M. (2012). Support vector machines for crop/weeds identification in maize fields. *Expert Systems with Applications*, 39(12), 11149-11155.

Guijarro, M., Pajares, G., Riomoros, I., Herrera, P. J., Burgos-Artizzu, X. P., & Ribeiro, A. (2011). Automatic segmentation of relevant textures in agricultural images. *Computers and Electronics in Agriculture*, 75(1), 75-83.

Haboudane, D., Miller, J. R., Pattey, E., Zarco-Tejada, P. J., & Strachan, I. B. (2004). Hyperspectral vegetation indices and novel algorithms for predicting green LAI of crop canopies: Modeling and validation in the context of precision agriculture. *Remote sensing of environment*, 90(3), 337-352.

Haboudane, D., Miller, J. R., Tremblay, N., Zarco-Tejada, P. J., & Dextraze, L. (2002). Integrated narrow-band vegetation indices for prediction of crop chlorophyll content for application to precision agriculture. *Remote sensing of environment*, 81(2-3), 416-426.

Hague, T., Tillett, N. D., & Wheeler, H. (2006). Automated crop and weed monitoring in widely spaced cereals. *Precision Agriculture*, 7, 21-32.

Huete, A. R. (1988). A soil-adjusted vegetation index (SAVI). *Remote sensing of environment*, 25(3), 295-309.

Hunt, E. R., Cavigelli, M., Daughtry, C. S., Mcmurtrey, J. E., & Walthall, C. L. (2005). Evaluation of digital photography from model aircraft for remote sensing of crop biomass and nitrogen status. *Precision Agriculture*, 6, 359-378.

Jasper, J., Reusch, S., & Link, A. (2009). Active sensing of the N status of wheat using optimized wavelength combination: impact of seed rate, variety and growth stage. In *Precision agriculture'09* (pp. 21-30). Wageningen Academic.

Jordan, C. F. (1969). Derivation of leaf‐area index from quality of light on the forest floor. *Ecology*, 50(4), 663-666.

Kataoka, T., Kaneko, T., Okamoto, H., & Hata, S. (2003, July). Crop growth estimation system using machine vision. In *Proceedings 2003 IEEE/ASME international conference on advanced intelligent mechatronics (AIM 2003)* (Vol. 2, pp. b1079-b1083). IEEE.

Le Maire, G., François, C., & Dufrene, E. (2004). Towards universal broad leaf chlorophyll indices using PROSPECT simulated database and hyperspectral reflectance measurements. *Remote sensing of environment*, 89(1), 1-28.

Louhaichi, M., Borman, M. M., & Johnson, D. E. (2001). Spatially located platform and aerial photography for documentation of grazing impacts on wheat. *Geocarto International*, 16(1), 65-70.

Merzlyak, M. N., Gitelson, A. A., Chivkunova, O. B., & Rakitin, V. Y. (1999). Non‐destructive optical detection of pigment changes during leaf senescence and fruit ripening. *Physiologia plantarum*, 106(1), 135-141.

Meyer, G. E., Hindman, T. W., & Laksmi, K. (1999, January). Machine vision detection parameters for plant species identification. In *Precision agriculture and biological quality* (Vol. 3543, pp. 327-335). SPIE.

Meyer, G. E., & Neto, J. C. (2008). Verification of color vegetation indices for automated crop imaging applications. *Computers and electronics in agriculture*, 63(2), 282-293.

Qi, J., Chehbouni, A., Huete, A. R., Kerr, Y. H., & Sorooshian, S. (1994). A modified soil adjusted vegetation index. *Remote sensing of environment*, 48(2), 119-126.

Richardson, A. J., & Wiegand, C. L. (1977). Distinguishing vegetation from soil background information. *Photogrammetric engineering and remote sensing*, 43(12), 1541-1552.

Rondeaux, G., Steven, M., & Baret, F. (1996). Optimization of soil-adjusted vegetation indices. *Remote sensing of environment*, 55(2), 95-107.

Roujean, J. L., & Breon, F. M. (1995). Estimating PAR absorbed by vegetation from bidirectional reflectance measurements. *Remote sensing of Environment*, 51(3), 375-384.

Sandham, L. A., & Zietsman, H. L. (1997). Surface temperature measurement from space: a case study in the south western cape of South Africa. *South African Journal of Enology and Viticulture* 18 (2):25-30.

Sripada, R. P., Heiniger, R. W., White, J. G., & Meijer, A. D. (2006). Aerial color infrared photography for determining early in‐season nitrogen requirements in corn. *Agronomy Journal*, 98(4), 968-977.

Tucker, C. J. (1979). Red and photographic infrared linear combinations for monitoring vegetation. *Remote sensing of Environment*, 8(2), 127-150.

Vincini, M., Frazzi, E. & D’Alessio, P. (2008). A broad-band leaf chlorophyll vegetation index at the canopy scale. *Precision Agriculture*, 9, 303-319.

Wang, W., Yao, X., Yao, X., Tian, Y., Liu, X., Ni, J., ... & Zhu, Y. (2012). Estimating leaf nitrogen concentration with three-band vegetation indices in rice and wheat. *Field Crops Research*, 129, 90-98.

Woebbecke, D. M., Meyer, G. E., Von Bargen, K., & Mortensen, D. A. (1995). Color indices for weed identification under various soil, residue, and lighting conditions. *Transactions of the ASAE*, 38(1), 259-269.

Zarco-Tejada, P. J., Berjón, A., Lopez-Lozano, R., Miller, J. R., Martín, P., Cachorro, V., ... & De Frutos, A. (2005). Assessing vineyard condition with hyperspectral indices: Leaf and canopy reflectance simulation in a row-structured discontinuous canopy. *Remote Sensing of Environment*, 99(3), 271-287.
