# Supplementary material for: Drought tolerance classification using unmanned aerial systems based on RGB and multispectral data
Source: Front Plant Sci. 2026 Jun 29;17:1853372. doi: 10.3389/fpls.2026.1853372 (PMC13357819; doi:10.3389/fpls.2026.1853372)
Supplement: Supplementary file 2 [file Table2.docx]

**Drought tolerance classification using unmanned aerial systems based on RGB and multispectral data**


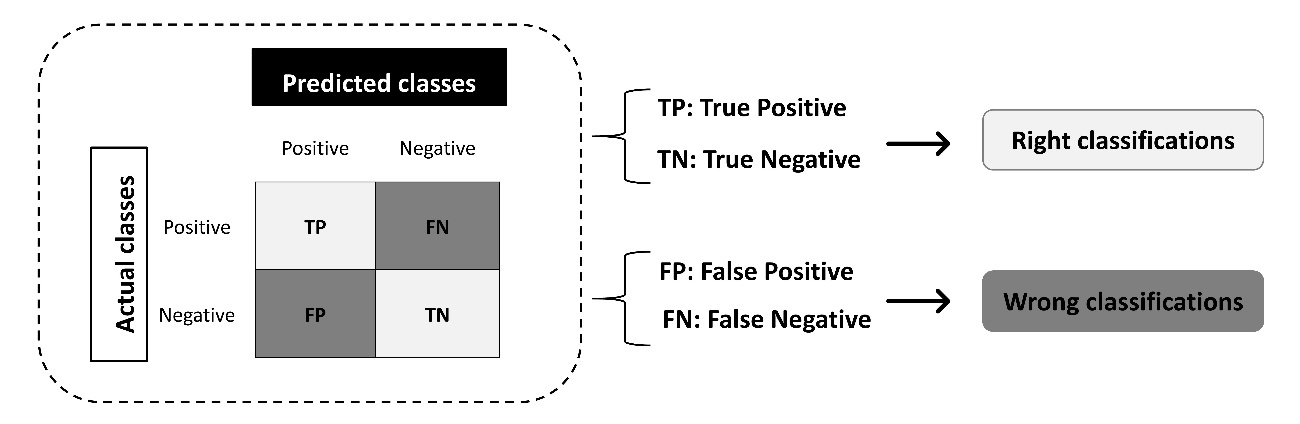


Supplementary Figure S1: Illustration of a confusion matrix with the four possible outcomes in a binary classification system, which serve as input to calculated metrics of classification efficiency.


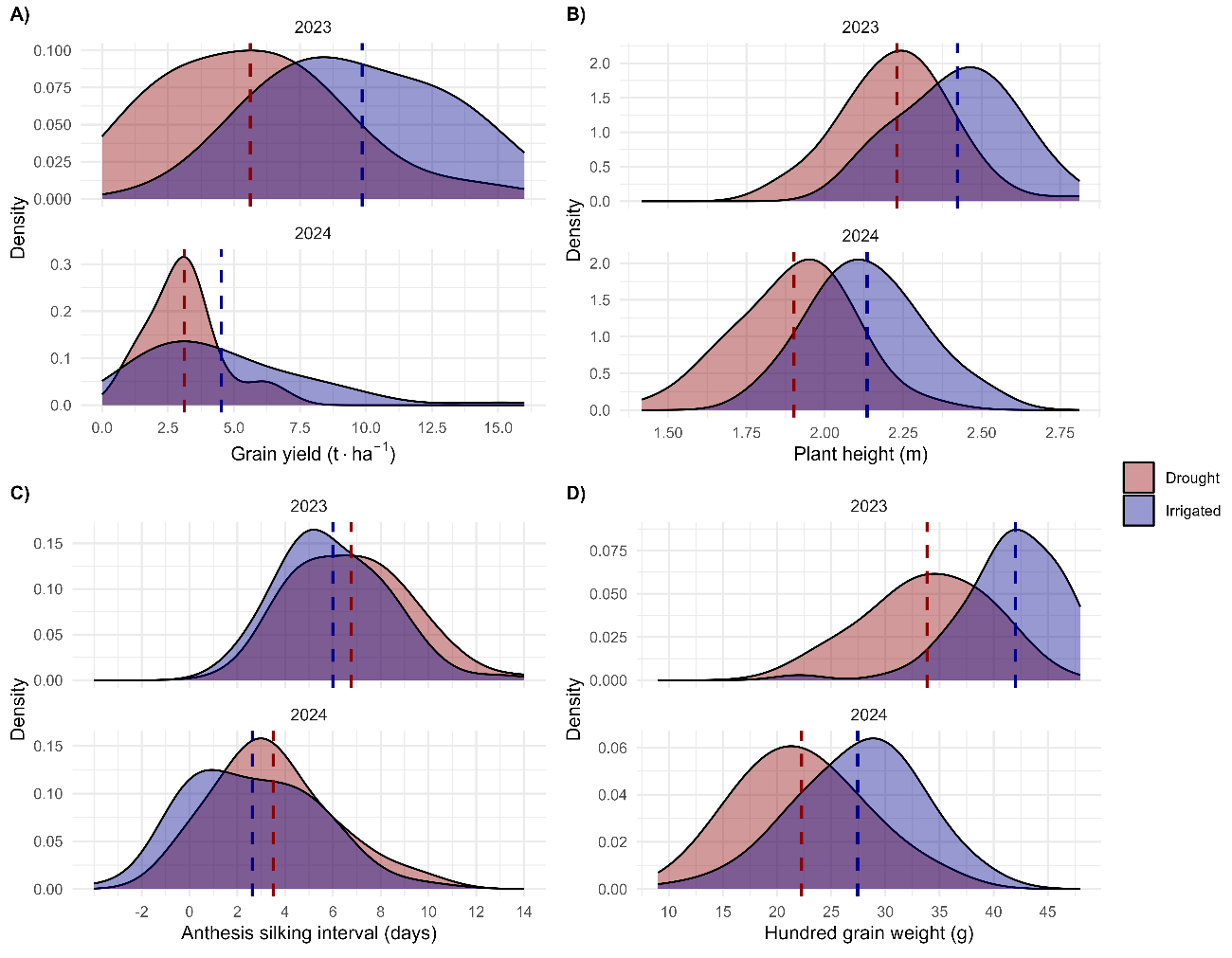


Supplementary Figure S2: Density curves of phenotypic data for the traits grain yield, plant height, anthesis-silking interval, and hundred-grain weight for both conditions within each year. The blue vertical dashed lines indicate the overall average for the trait under irrigated conditions, and the red vertical dashed lines indicate the overall average under drought conditions.


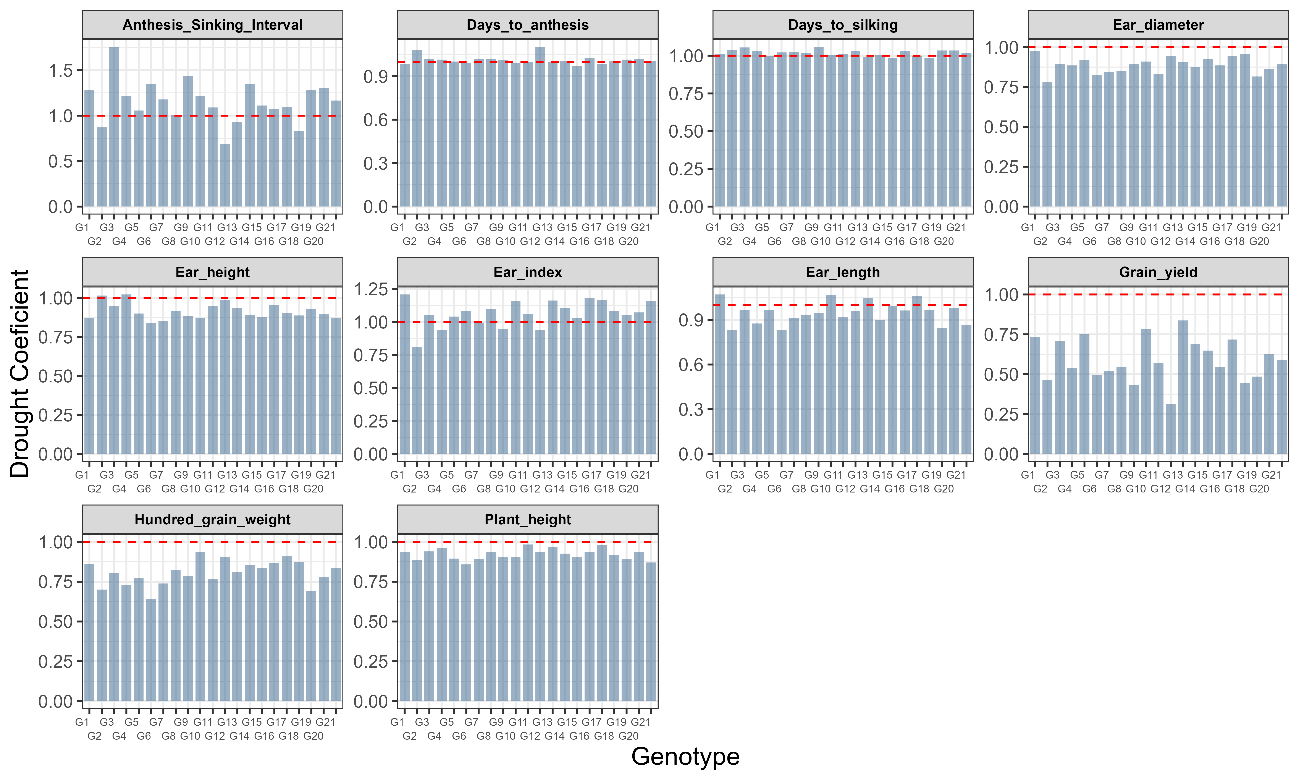


Supplementary Figure S3: Drought coefficients for each genotype and trait in 2023. The red horizontal dashed lines for each trait indicate a drought coefficient value of 1, meaning performance under drought was equal to that under irrigated conditions.


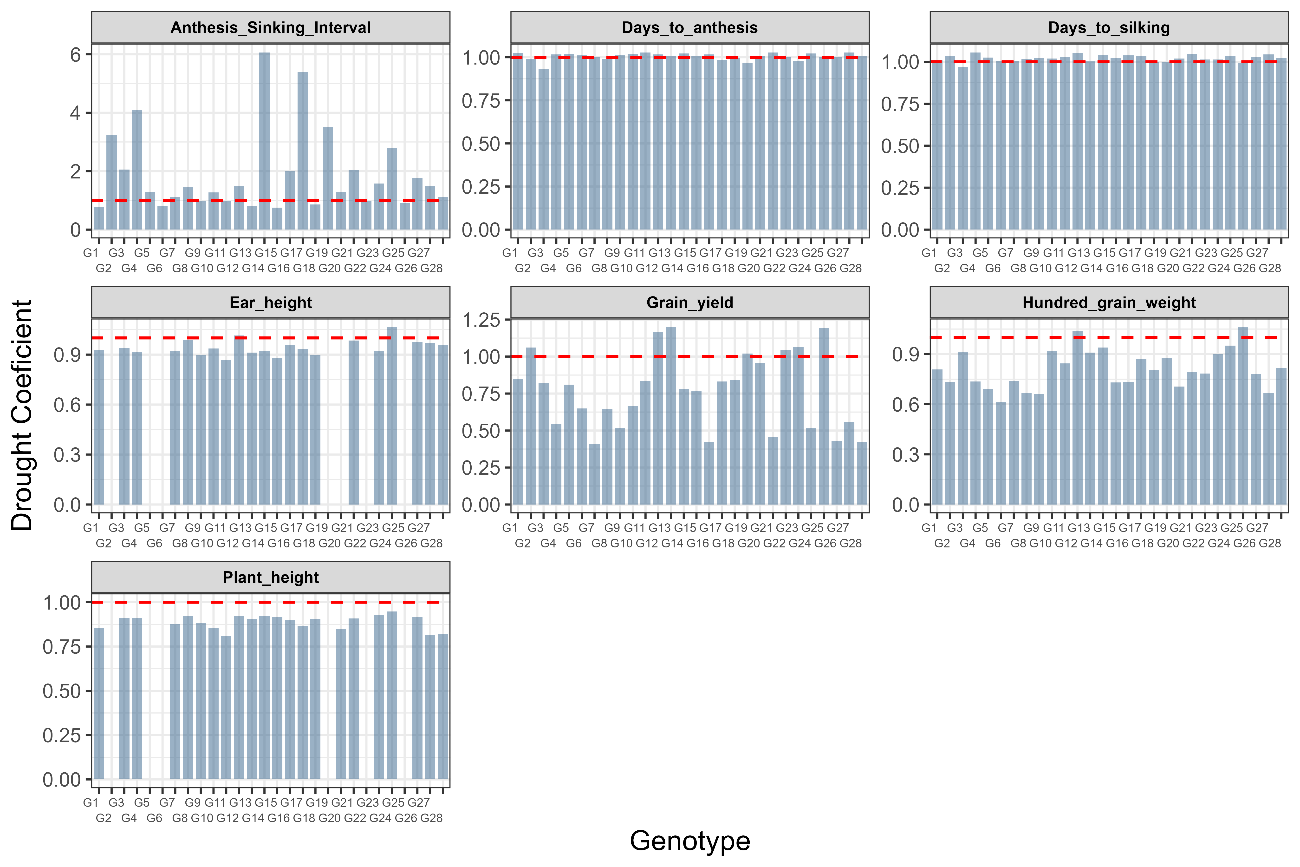
 Supplementary Figure S4: Drought coefficients for each genotype and trait in 2024. The red horizontal dashed lines for each trait indicate a drought coefficient of 1, meaning that performance under drought was the same as in irrigated conditions. The white spaces in the plots of ear and plant height are due to missing data for at least one of the trials (irrigated or drought) for the genotypes, which prevented calculation of the drought coefficient.


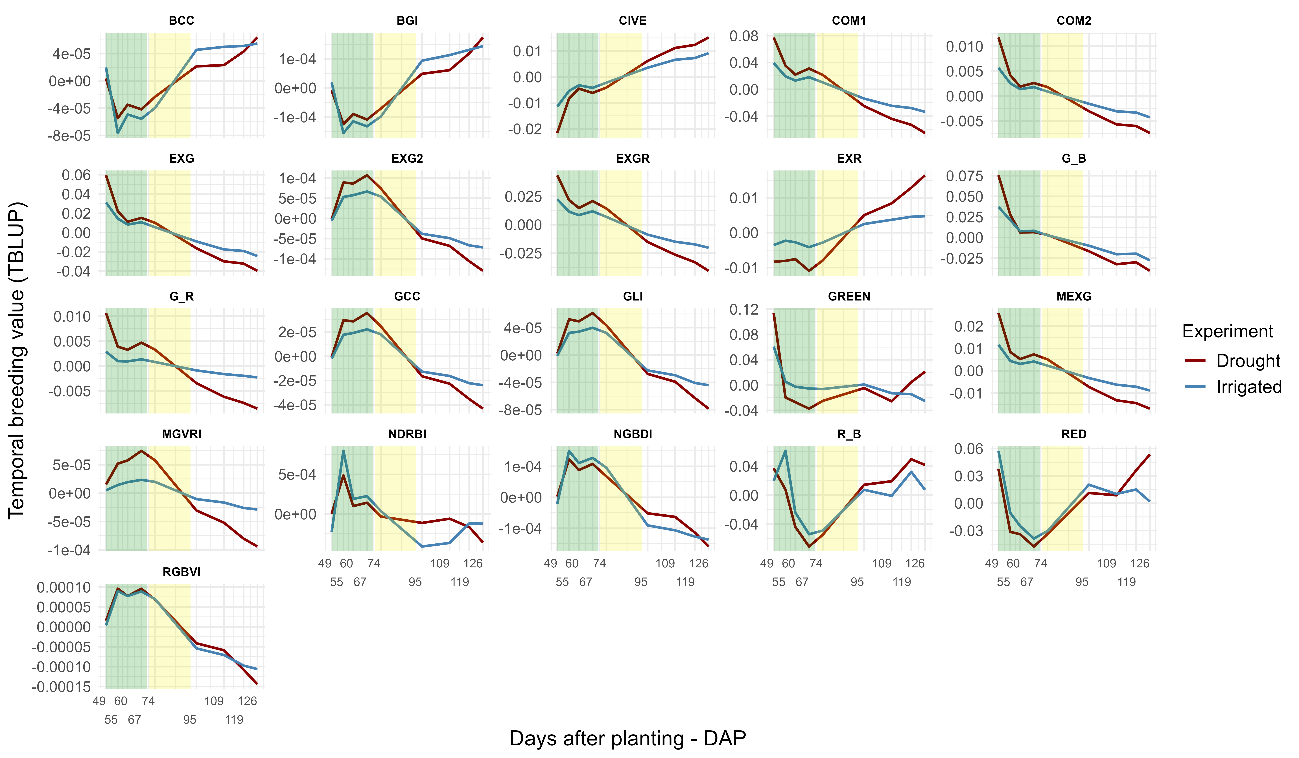
 Supplementary Figure S5: Temporal breeding values (TBLUPs) for the significant RGB vegetation indices in 2023 for each trial along the flights of phenotyping. Each Y-axis shows the unique ranges of TBLUPs, while the X-axis shows the flight dates as days after planting (DAP), consistent across all indices. The green and yellow shaded areas delimit the vegetative and flowering periods (70 to 92 DAP), respectively, and assist in visualizing each stage of the crop cycle.


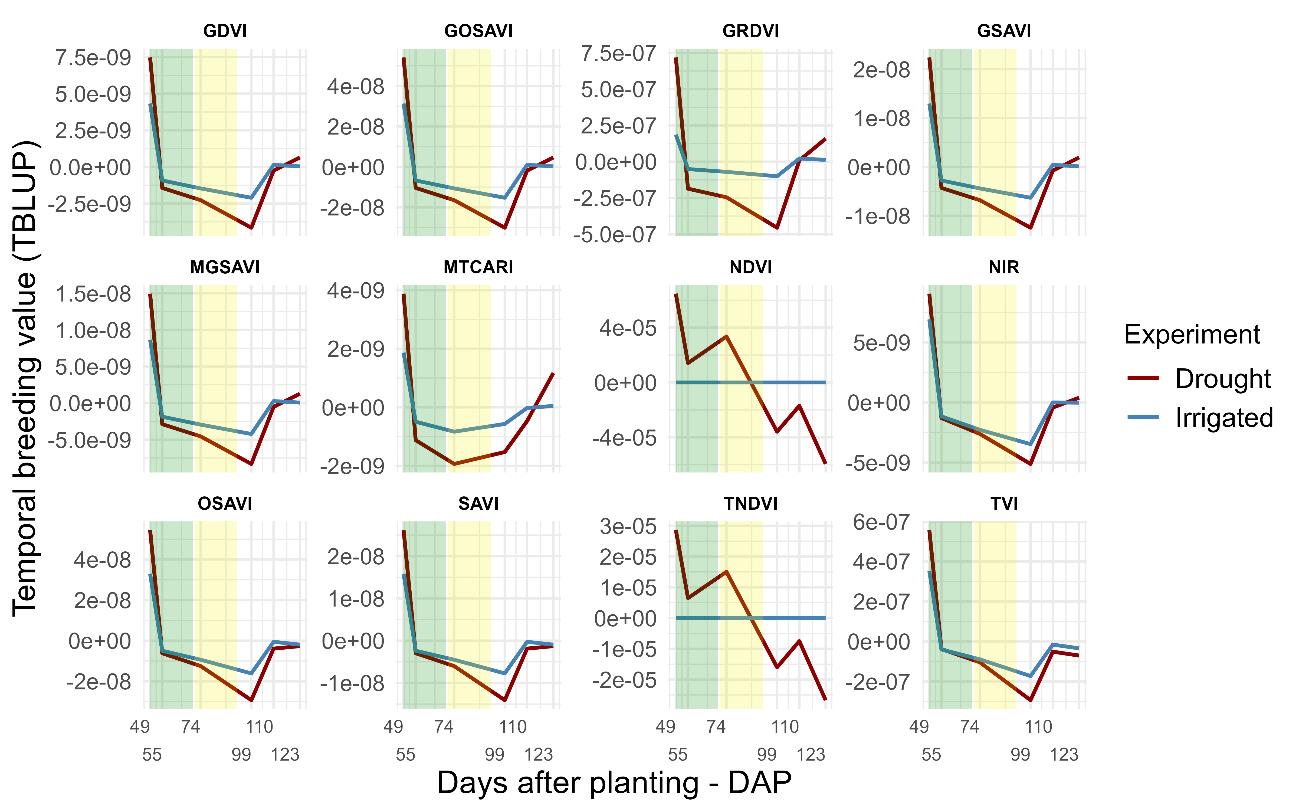


Supplementary Figure S6: Temporal breeding values (TBLUPs) for the significant multispectral vegetation indices in 2023 for each trial along the flights of phenotyping. Each Y-axis shows the unique ranges of TBLUPs, while the X-axis indicates the flight dates as days after planting (DAP), consistent across all indices. The blue lines for the indices TNDVI and NDVI appear to be zero because the difference between their values and the values under drought (red lines) was very large. Therefore, the same Y-axis scale for both conditions limited the visibility of TBLUPs under irrigated trials for these two indices. The green and yellow shaded areas delimit the vegetative and flowering periods (70 to 92 DAP), respectively, and help to visualize each stage of the crop cycle.


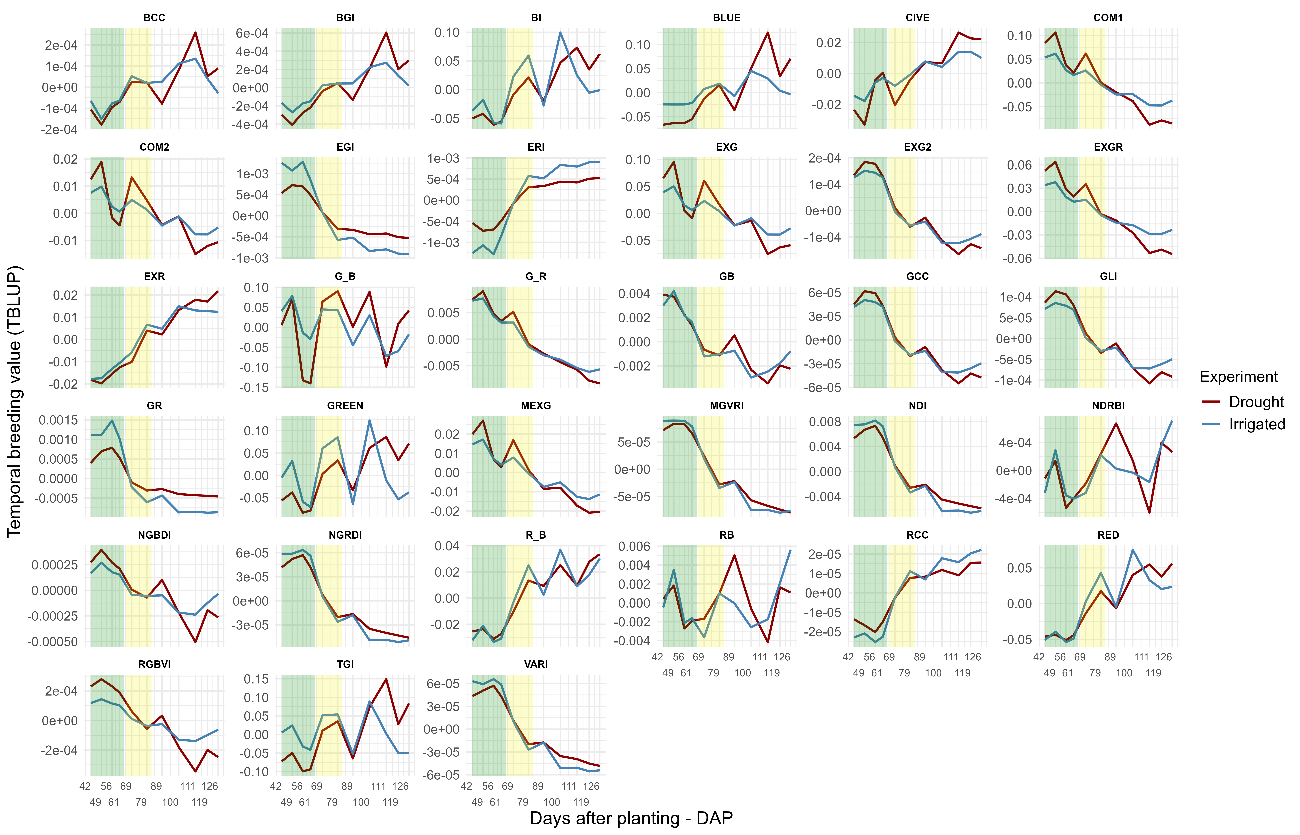


Supplementary Figure S7: Temporal breeding values (TBLUPs) for the significant RGB vegetation indices in 2024 for each trial along the phenotyping flights. Each Y-axis shows the specific ranges of TBLUPs, while the X-axis displays the flight dates in days after planting (DAP), consistent across all indices. The green and yellow shaded areas delineate the vegetative and flowering periods (65 to 82 DAP), respectively, aiding visualization within each stage of the crop cycle.


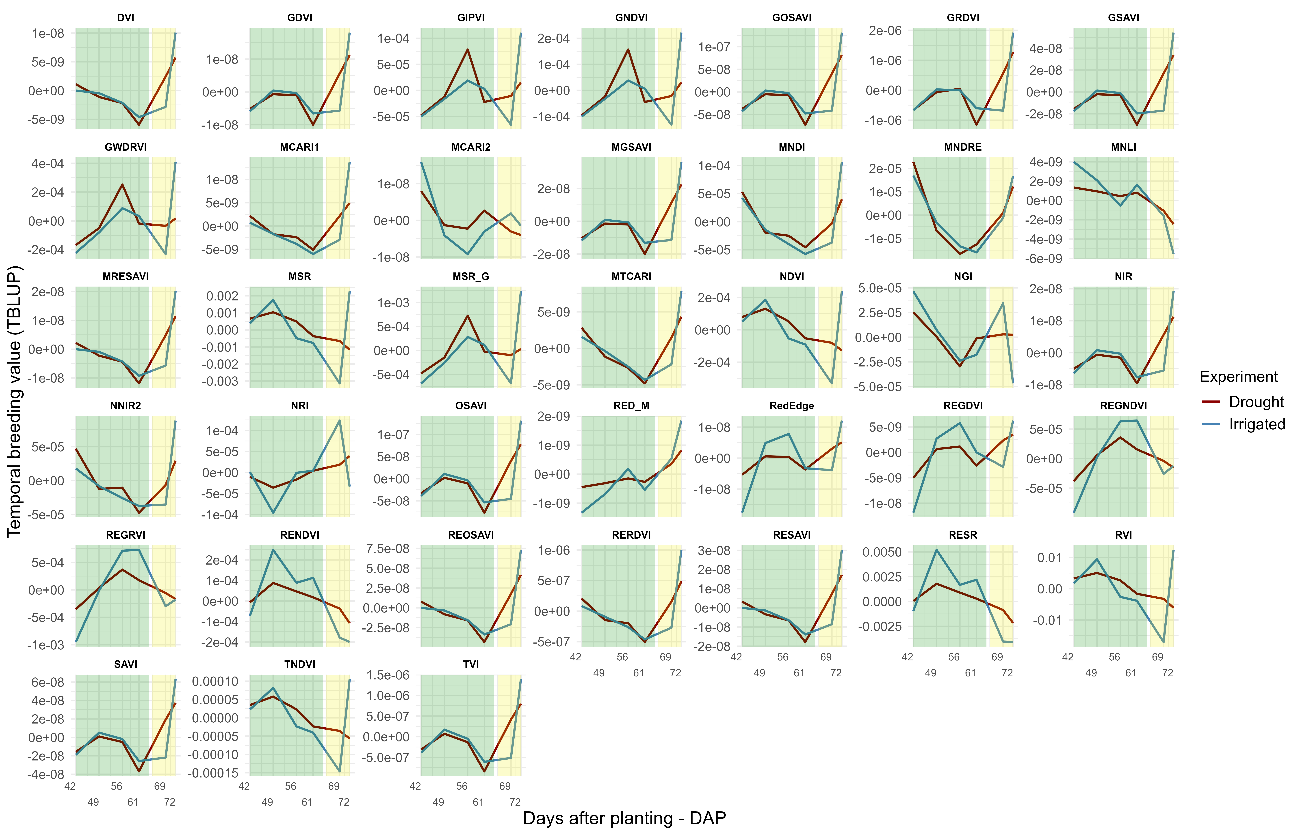
 Supplementary Figure S8: Temporal breeding values (TBLUPs) for the significant multispectral vegetation indices in 2024 for each trial over the course of phenotyping flights. Each Y-axis shows the specific range of TBLUPs, while the X-axis represents the flight dates in days after planting (DAP), consistent across all indices. The green and yellow shaded areas mark the vegetative and flowering periods (65 to 72 DAP), respectively, helping to visualize each stage of the crop cycle.


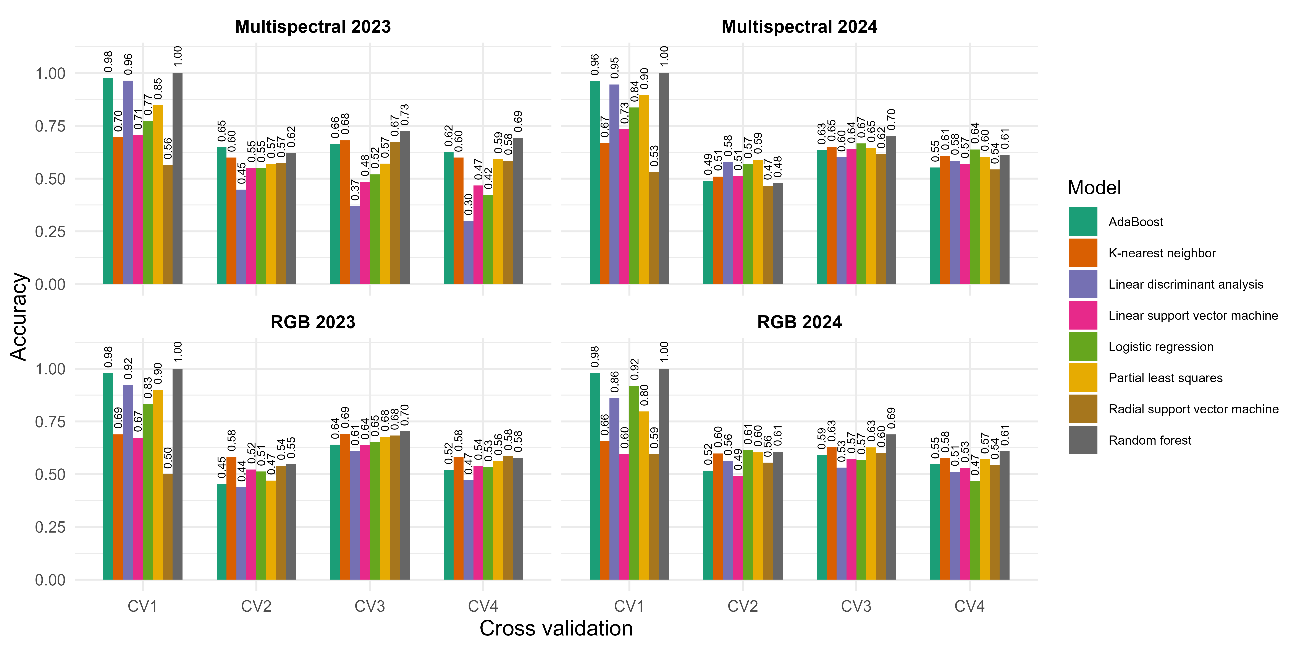
 Supplementary Figure S9: Accuracy of drought tolerance classification (Y axis) for each combination of sensor and year, across the four cross-validation schemes (X axis) and the eight machine learning models tested, training the models on irrigated trials. CV1 and CV2 refer to the classification of tested and untested genotypes in the observed environment (irrigated), respectively. CV3 and CV4 refer to the classification of tested and untested genotypes in the unobserved environment (drought), respectively.


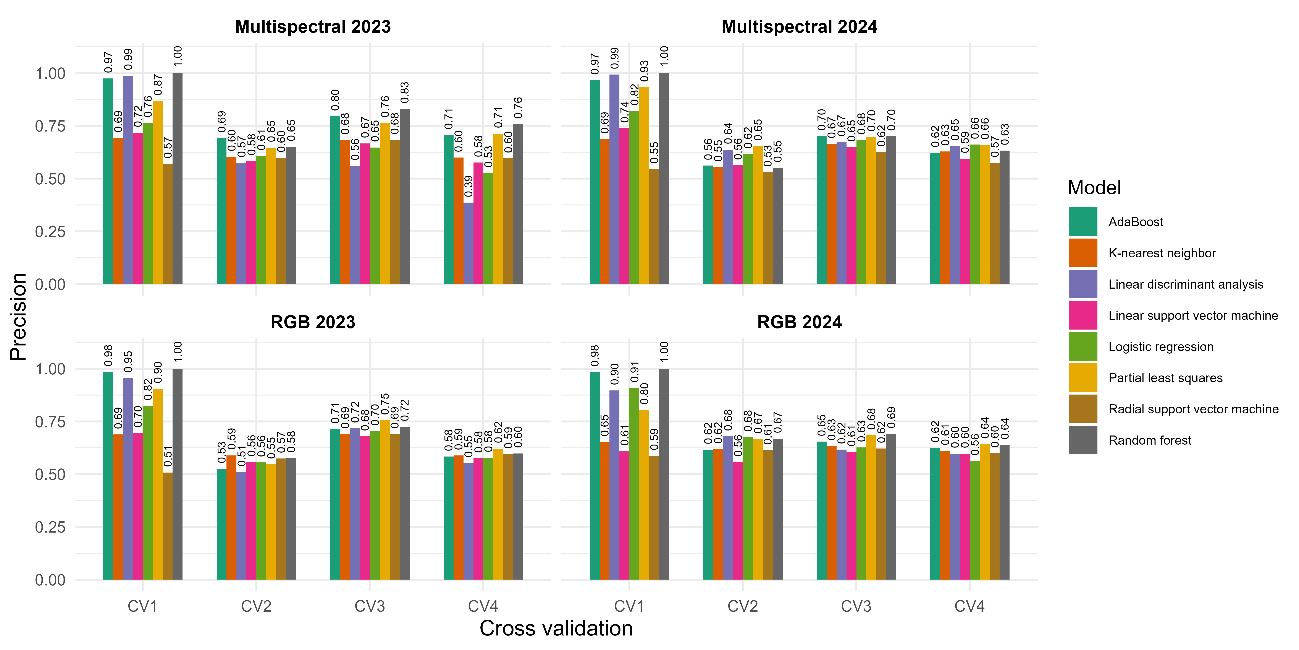
 Supplementary Figure S10: Precision of drought tolerance classification (Y axis) for each combination of sensor and year, across the four cross-validation schemes (X axis) and the eight machine learning models tested, training the models in irrigated trials. CV1 and CV2 represent the classification of tested and untested genotypes in the observed (irrigated) environment, respectively. CV3 and CV4 represent the classification of tested and untested genotypes in the unobserved (drought) environment, respectively.


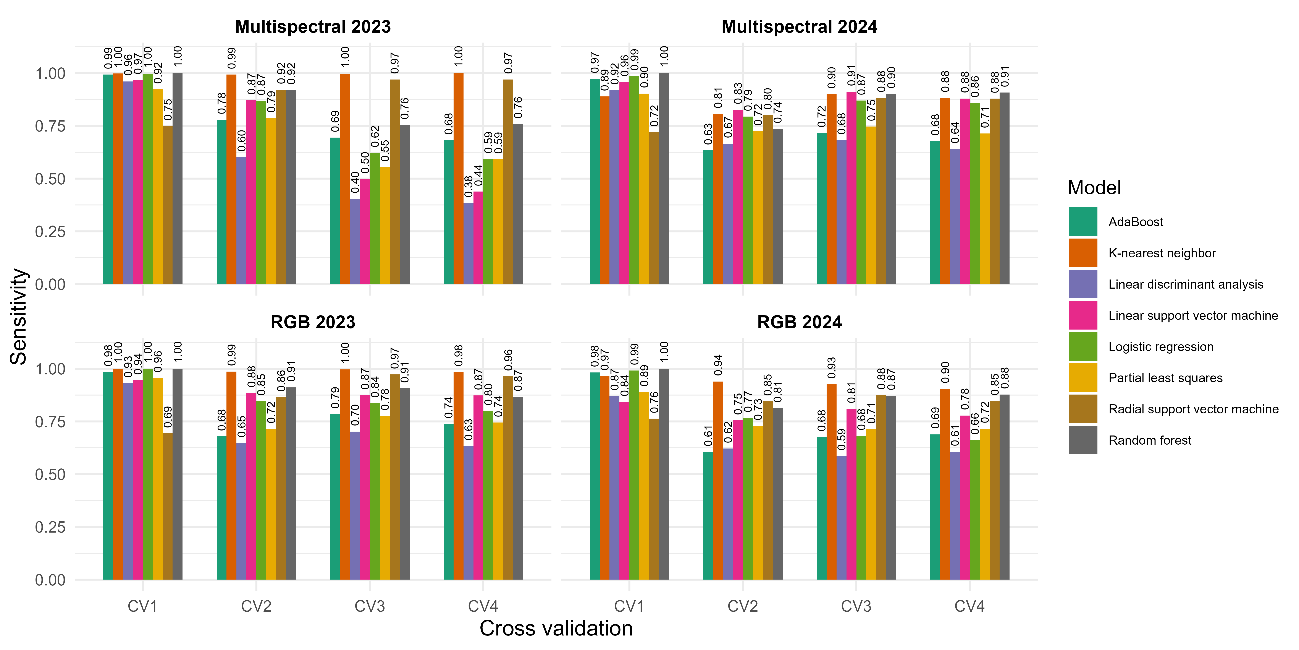
 Supplementary Figure S11: Sensitivity of drought tolerance classification (Y axis) for each combination of sensor and year, across the four cross-validation schemes (X axis) and the eight machine learning models tested, with models trained on irrigated trials. CV1 and CV2 represent classification of tested and untested genotypes in the observed environment (irrigated), respectively. CV3 and CV4 represent classification of tested and untested genotypes in the unobserved environment (drought), respectively.


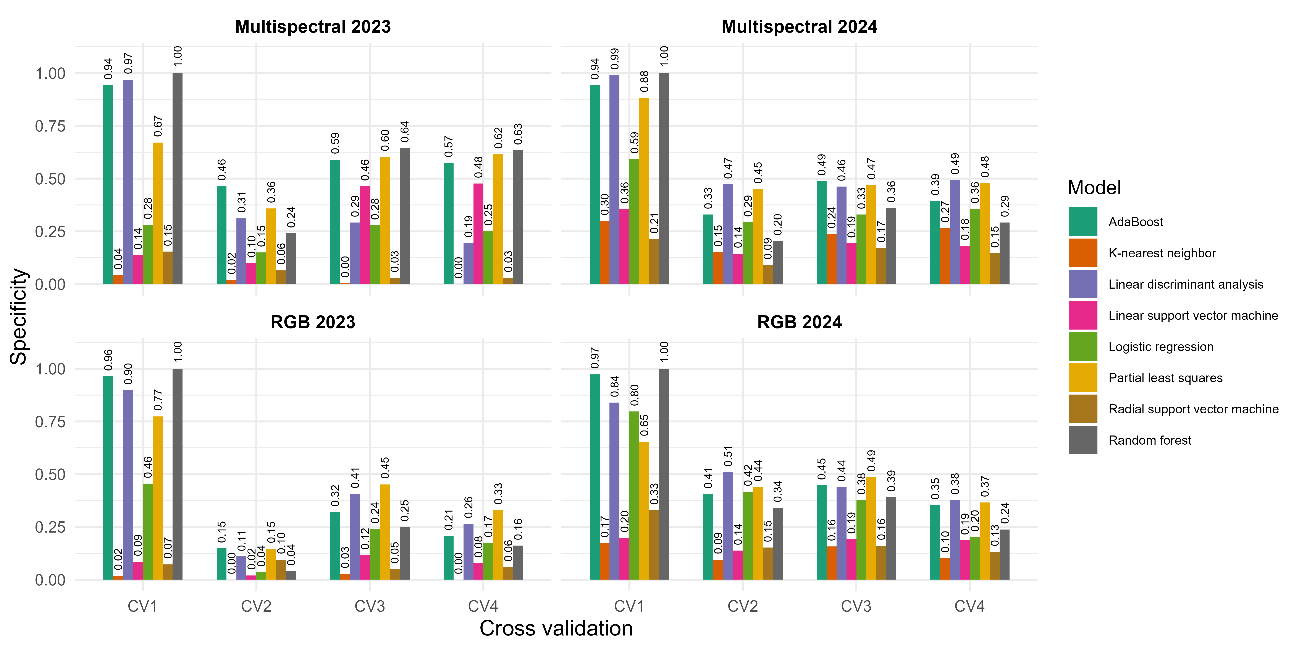
 Supplementary Figure S12: Specificity of drought tolerance classification (Y axis) for each combination of sensor and year, across the four cross-validation schemes (X axis) and the eight machine learning models tested, trained on irrigated trials. CV1 and CV2 indicate classification of tested and untested genotypes in the observed environment (irrigated), respectively. CV3 and CV4 indicate classification of tested and untested genotypes in the unobserved environment (drought), respectively.


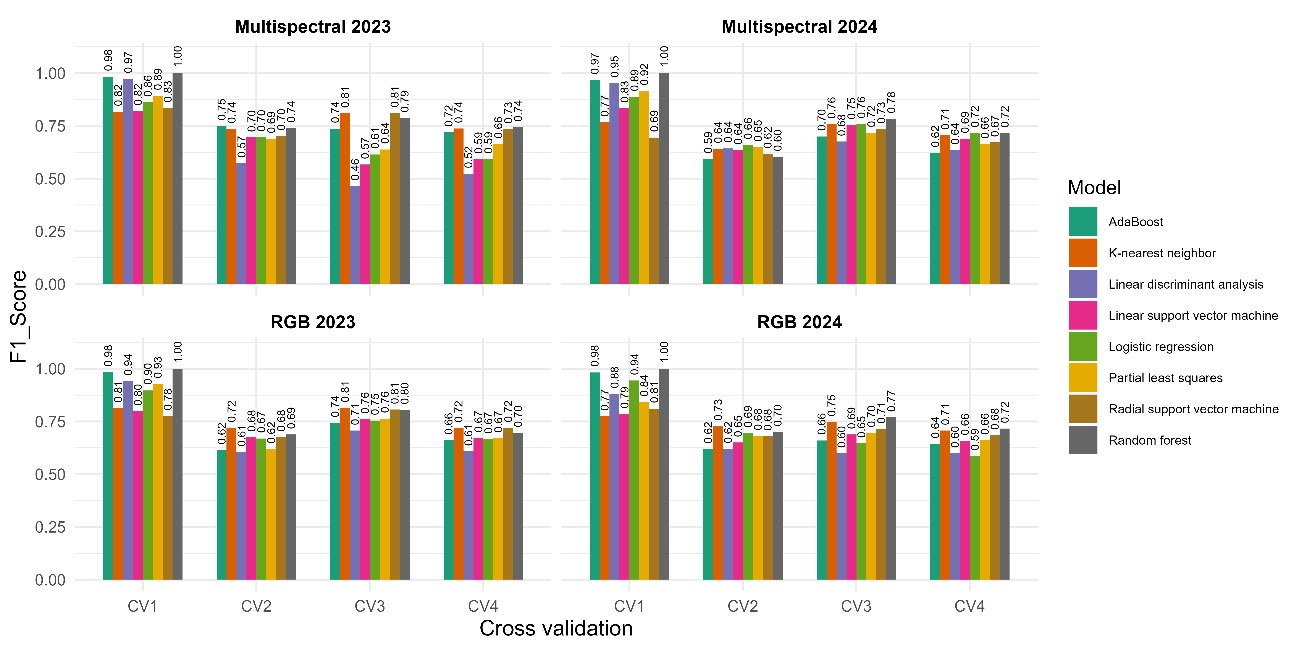
 Supplementary Figure S13: F1-score of drought tolerance classification (Y axis) for each combination of sensor and year, across the four cross-validation schemes (X axis) and the eight machine learning models tested, training the models in irrigated trials. CV1 and CV2 represent classification of tested and untested genotypes in the observed environment (irrigated), respectively. CV3 and CV4 represent classification of tested and untested genotypes in the unobserved environment (drought), respectively.


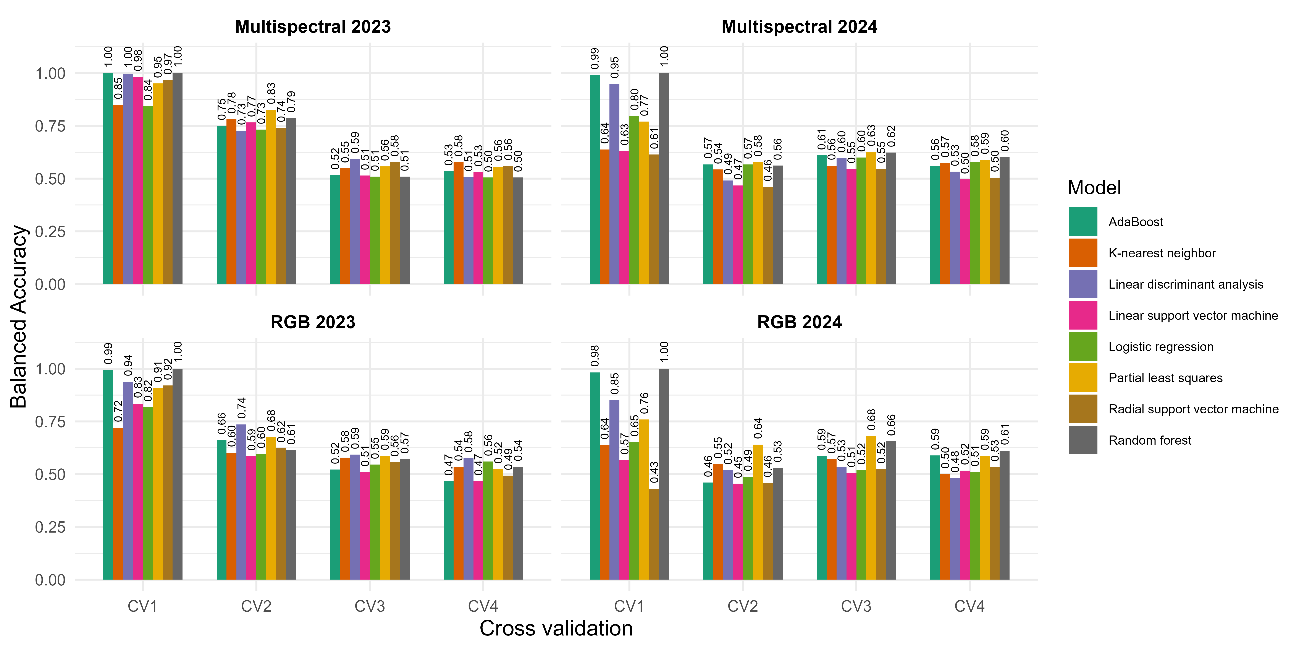


Supplementary Figure S14: Balanced accuracy of drought tolerance classification (Y axis) for each combination of sensor and year, across the four cross-validation schemes (X axis) and the eight machine learning models tested, training the models in drought trials. CV1 and CV2 represent classification of tested and untested genotypes in the observed environment (drought), respectively. CV3 and CV4 represent classification of tested and untested genotypes in the unobserved environment (irrigated), respectively.
